# The *Gossypium hirsutum* TIR‐NBS‐LRR gene *GhDSC1 *mediates resistance against Verticillium wilt

**DOI:** 10.1111/mpp.12797

**Published:** 2019-04-08

**Authors:** Ting‐Gang Li, Bao‐Li Wang, Chun‐Mei Yin, Dan‐Dan Zhang, Dan Wang, Jian Song, Lei Zhou, Zhi‐Qiang Kong, Steven J. Klosterman, Jun‐Jiao Li, Sabiu Adamu, Ting‐Li Liu, Krishna V. Subbarao, Jie‐Yin Chen, Xiao‐Feng Dai

**Affiliations:** ^1^ Laboratory of Cotton Disease, Institute of Food Science and Technology Chinese Academy of Agricultural Sciences Beijing 100193 China; ^2^ Key Laboratory of Agro‐products Quality and Safety Control in Storage and Transport Process, Ministry of Agriculture Beijing 100193 China; ^3^ United States Department of Agriculture Agricultural Research Service Salinas California USA; ^4^ Provincial Key Laboratory of Agrobiology Jiangsu Academy of Agricultural Sciences Nanjing Jiangsu 210014 China; ^5^ Department of Plant Pathology University of California, Davis, c/o United States Agricultural Research Station Salinas California USA

**Keywords:** calmodulin binding transcription activator (CAMTA), *Gossypium hirsutum*, nonsynonymous mutation, TIR‐NBS‐LRR gene, Verticillium wilt

## Abstract

Improving genetic resistance is a preferred method to manage Verticillium wilt of cotton and other hosts. Identifying host resistance is difficult because of the dearth of resistance genes against this pathogen. Previously, a novel candidate gene involved in Verticillium wilt resistance was identified by a genome‐wide association study using a panel of *Gossypium hirsutum* accessions. In this study, we cloned the candidate resistance gene from cotton that encodes a protein sharing homology with the TIR‐NBS‐LRR receptor‐like defence protein *DSC1* in *Arabidopsis thaliana* (hereafter named *GhDSC1*). *GhDSC1 *expressed at higher levels in response to Verticillium wilt and jasmonic acid (JA) treatment in resistant cotton cultivars as compared to susceptible cultivars and its product was localized to nucleus. The transfer of *GhDSC1 *to *Arabidopsis* conferred Verticillium resistance in an *A. thaliana* *dsc1* mutant. This resistance response was associated with reactive oxygen species (ROS) accumulation and increased expression of JA‐signalling‐related genes. Furthermore, the expression of *GhDSC1* in response to Verticillium wilt and JA signalling in *A. thaliana* displayed expression patterns similar to *GhCAMTA3* in cotton under identical conditions, suggesting a coordinated *DSC1* and *CAMTA3 *response in *A. thaliana* to Verticillium wilt. Analyses of *GhDSC1 *sequence polymorphism revealed a single nucleotide polymorphism (SNP) difference between resistant and susceptible cotton accessions, within the P‐loop motif encoded by *GhDSC1*. This SNP difference causes ineffective activation of defence response in susceptible cultivars. These results demonstrated that *GhDSC1* confers Verticillium resistance in the model plant system of *A. thaliana*, and therefore represents a suitable candidate for the genetic engineering of Verticillium wilt resistance in cotton.

## Introduction

Resistance (*R*) genes are key components of genetic interactions between plants and pathogens and are known to activate immunity/resistance responses against pathogen invasion (Chisholm *et al*., 2006; Dodds and Rathjen, 2010; Jones and Dangl, 2006). The common motifs of *R *gene products include a nucleotide‐blinding site (NBS), leucine‐rich repeat (LRR), Drosophila Toll domain, the mammalian interleukin‐1 receptor (TIR), coiled‐coil structure (CC), transmembrane domain (TM), and serine/threonine protein kinase domain (PK), which can be grouped into several typical families of TIR‐NBS‐LRRs (TNL), CC‐NBS‐LRR (CNL), nucleotide‐binding site leucine‐rich repeat (NBS‐LRR), LRR‐TM, LRR‐TM‐PK. (Joshi and Nayak, 2011; Martin *et al*., 2003; McHale *et al*., 2006). Most disease resistance genes in plants encode NBS‐LRR proteins. The genes encoding NBS‐LRR proteins can be subdivided into the functionally distinct TIR‐domain‐containing (TNL) and CC‐domain‐containing (CNL) subfamilies (McHale *et al*., 2006). For example, 149 NBS‐LRR proteins are encoded in the genome of *Arabidopsis thaliana* (Meyers *et al*., 2003).

To date, numerous NBS‐LRR proteins with roles in mediating plant disease resistance have been identified (Anderson *et al*., 1997; Ellis *et al*., 1999; Feuillet *et al*., 2003; Hinsch and Staskawicz, 1996; Li *et al*., 2017; Periyannan *et al*., 2013; Sanseverino *et al*., 2012; Shen *et al*., 2007; Wang *et al*., 2015; Whitham *et al*., 1994; Zhu *et al*., 2017). NBS‐LRR proteins generally are composed of tripartite domain architectures, an N‐terminal response domain involved in downstream signalling (CC or TIR are examples), a central molecular switch domain (NB‐ARC, a nucleotide‐binding adaptor shared by the mammalian apoptosis regulator Apaf1, and the *Caenorhabditis elegans* apoptosis regulator CED4), and a C‐terminal sensor domain‐containing LRRs (Collier and Moffett, 2009; Van der Biezen and Jones, 1998; Maekawa *et al*., 2011; Meyers *et al*., 2003; Qi and Innes, 2013).

The simplest model for NBS‐LRR protein function is that they act as receptors that bind effector proteins secreted by pathogens, but only a few such direct interactions have been characterized (Deslandes *et al*., 2003; Jia *et al*., 2000). In an alternative model, the ‘guard hypothesis’ predicts that NBS‐LRR proteins act by monitoring the status of plant proteins targeted by pathogen effectors, and that modification of this target by the effector results in the activation of the R protein, which triggers disease resistance in the host (Dangl and Jones, 2001; Van der Biezen and Jones, 1998). For instance, *RPM1* (CC‐NBS‐LRR) detects phosphorylation of *RPM1*‐Interacting Protein 4 (*RIN4*) by the pathogen effectors from *Pseudomonas syringae*, and this modification elicits the resistance response (Mackey *et al*., 2002). In the majority of these interactions, it is the N‐terminus of the TIR or CC domain that is primarily responsible for the interaction with the downstream signalling partner, while the NBS is mainly involved in adenosine triphosphate (ATP) hydrolysis (the ADP [adenosine diphosphate] bound state represents the ‘off’ and the ATP the ‘on’ state) and release of the signalling. The LRR domain is responsible for interaction with signalling partners for the activation or interaction with the upstream activator (Belkhadir *et al*., 2004; McHale *et al*., 2006).

In *Gossypium* spp., *R*‐genes have been predicted and systematically compared using common motifs (Chen *et al*., 2015). The diploid *Gossypium raimondii *genome encodes more than 1000 resistance gene analogues (RGAs) and most of these genes cluster in homology groups based on high levels of protein sequence similarity (Chen *et al*., 2015). Amongst these, more than 300 *G. raimondii* RGAs encode NBS domains, largely of the CC‐NBS and CC‐NBS‐LRR subgroups (Paterson *et al*., 2012; Wei *et al*., 2013). Systematic analysis and comparison of NBS domain‐containing proteins in *G. raimondii* revealed 163 NBS genes that contain all five conserved motifs (P‐loop, Kinase2, Kinase3, GLPL and MHDL) (Paterson *et al*., 2012; Wei *et al*., 2013), and the disease resistance QTL (quantitative trait loci) were adjacent to the NBS‐encoding genes (Wei *et al*., 2013).

Verticillium wilt of cotton, caused by the soil‐borne fungus *Verticillium dahliae*, is a devastating disease that results in major losses in yield and boll quality (Xu *et al*., 2011b). Developing resistance in cotton cultivars is considered the optimal method to manage Verticillium wilt, which makes identifying Verticillium wilt resistance genes in cotton germplasm and incorporating them into elite cultivars a priority. The *Ve1 *gene (encoding a receptor‐like protein, LRR‐TM) mediates defence against *V. dahliae* race 1 strains in tomato (Fradin *et al*., 2009), and several similar genes have been identified in cotton using candidate homologues, including *GbVe*, *GbVe1*, *Gbvdr5*, *GbaVd1 *and *GbaVd2 *(Chen *et al*., 2017; Yang *et al*., 2015; Zhang *et al*., 2011, 2012). However, the *Ve1* homologue does not provide adequate resistance in cotton since most of the *V. dahliae *strains from cotton lack the corresponding *Ave1* effector (Song *et al*., 2018). Several other types of genes have been characterized that contribute to defence responses against Verticillium wilt, including *GbCAD1* and *GbSSI2* (Gao *et al*., 2013), *GbRLK *(Zhao *et al*., 2013), *GbSTK* (Zhang *et al*., 2013b), *GbTLP1* (Munis *et al*., 2010), *GbSBT1* (Duan *et al*., 2016), *GhPAO* (Mo *et al*., 2015) and *GbNRX1* (Li *et al*., 2016). However, NBS domain‐containing proteins involved in Verticillium wilt resistance have rarely been reported.

Comparative genomics suggested that expansion and contraction in the numbers of NBS‐encoding genes has altered Verticillium wilt resistance in *G. raimondii *(nearly immune to the *V. dahliae*) and *Gossypium arboretum *(highly susceptible to *V. dahliae*) (Li *et al*., 2014). Transcriptome analysis revealed that the NBS‐encoding genes were significantly up‐regulated during infection by *V. dahliae* (Chen *et al*., 2015; Li *et al*., 2014; Xu *et al*., 2011b; Zhang *et al*., 2013a). Moreover, the NBS‐encoding genes *GbRVd *and *GbaNA1 *contribute to defence responses against Verticillium wilt (Li *et al*., 2018a; Yang *et al*., 2016). In our previous study, a Verticillium wilt resistance locus was determined by genome‐wide association study (GWAS) using a panel of 299 *G. hirsutum *accessions and identified a novel candidate gene CG02 that encodes a TIR‐NBS‐LRR protein (Li *et al*., 2017), which also shares affinity to the known resistance gene *DSC1* from *A. thaliana* (Lolle *et al*., 2017) (and the gene is henceforth referred to as *GhDSC1* in this study). Since the most widely deployed cultivar of *G. hirsutum* appears to lack genetic resistance against *V. dahliae*, and few of NBS‐encoding genes have been identified to confer Verticillium wilt resistance (Cai *et al*., 2009; Zhang *et al*., 2011), the pool of these candidate genes could be an important resource to develop resistance in cotton.

The main objectives of the current study were to: 1) investigate the conserved structure of *GhDSC1*‐encoded proteins and their subcellular localization; 2) explore the relationship between plant hormones and defence responses mediated by *GhDSC1*; 3) study the role of the *GhDSC1* in Verticillium wilt resistance of *A. thaliana* transgenic lines using the *DSC1* orthologous mutants of *A. thaliana*; 4) explore the defence responses mediated by *GhDSC1*; and 5) investigate the allelic divergence of *GhDSC1* between resistant and susceptible accessions of *G. hirsutum*.

## Results

### *GhDSC1 *encodes a TIR‐NBS‐LRR protein

**Figure 1:**
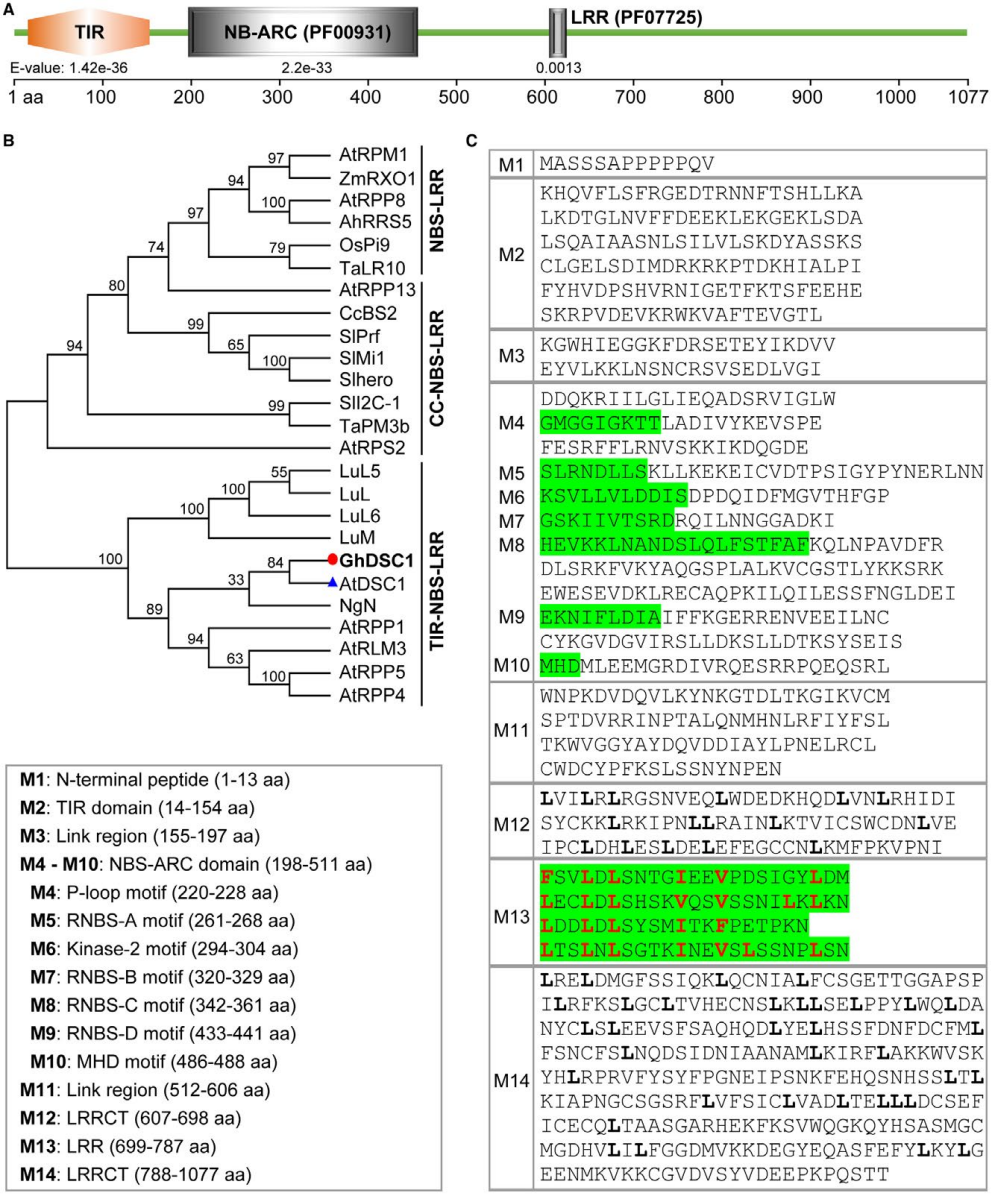
*GhDSC1* from *Gossypium hirsutum* encodes a TIR‐NBS‐LRR protein. (A) Peptide domain prediction in *GhDSC1*. Conserved domains of *GhDSC1 *were predicted using the web‐based programme SMART (http://smart.embl-heidelberg.de/). TIR, Toll ‐ interleukin 1 ‐ resistance; NB‐ARC, nucleotide‐binding adaptor shared APAF‐1, R proteins and CED‐4; LRR, leucine‐rich repeat. *E*‐value represents the confidence of the predicted domains. (B) Phylogenetic tree constructed using *GhDSC1* and known NBS‐LRR resistant proteins. The phylogeny was constructed by Mega 6.0, using maximum‐likelihood (Parameters: 1000 bootstraps, Jones‐Taylor‐Thornton model). *GhDSC1* and the closest orthologue *DSC1 *(named *AtDSC1* in this figure) from *A. thaliana* were labelled with a red dot and blue triangle, respectively. The known NBS‐LRR proteins include *A. thaliana AtRPM1 *(GeneBank: AGC12590.1), *AtRPP13* (GeneBank: AAF42831.1), *AtRPP8* (GeneBank: BAC67706.1), *AtDSC1* (GeneBank: NP_192938.1), *AtRPP1 *(GeneBank: NP_190034.2), *AtRLM3 *(GeneBank: AEE83835.1), *AtRPP4* (GeneBank: AAM18462.1), *AtRPP5* (GeneBank: AAF08790.1) *AtRPS2* (GeneBank: AAM90858.1); *Solanum lycopersicum SlPrf* (GeneBank: AAF76312.1), *SlMi1* (GeneBank: AAC97933.1), *SlI2C‐1* (GeneBank: AAB63274.1), *Slhero* (GeneBank: CAD29728.1); *Linum usitatissimum LuL6* (GeneBank: AAA91022.1), *LuL* (GeneBank: AAD25969.1), *LuM* (GeneBank: AAB47618.1), *LuL5* (GeneBank: AAD25972.1); *Nicotiana glutinosa NgN* (GeneBank: AAA50763.1); *Capsicum chacoense CcBS2* (GeneBank: AAF09256.1); *Triticum aestivum TaPM3b* (GeneBank: AAQ96158.1), *TaLR10* (GeneBank: AAQ01784.1); *Zea mays ZmRXO1* (GeneBank: AAX31149.1); *Oryza sativa OsPi9* (GeneBank: ABB88855.1); *Arac hishypogaea AhRRS5* (Zhang et al., 2017). (C) Analysis of GhDSC1 sequence characteristics. Sequence characteristics were drawn by the multiple sequence alignment of the *GhDSC1* to known TIR‐NBS‐LRR proteins. The amino acids represented in green indicate conserved motifs (labelled in M1–M14); search of LRRs (L, M and N regions) was conducted using the web‐based programme LRRfinder (http://www.lrrfinder.com/lrrfinder.php), L and N regions were predicted as LRRCT (PF01463: Leucine‐rich‐repeat C‐terminal domain). The leucine (L) residues were marked in bold font. Four LRRs were predicted and the L residues and similar hydrophobic amino acid residues were marked in red font.

We previously identified a candidate gene, CG02 that encodes a NBS‐LRR protein, contributing to Verticillium wilt resistance by virus‐induced gene silencing (VIGS) in upland cotton (Li *et al*., 2017) and was subsequently named *GhDSC1*. To further investigate the role of *GhDSC1* in Verticillium wilt resistance, DNA or cDNA sequences were identified from the resistant *G. hirsutum *cv. Zhongzhimian No. 2 at the genomic and transcriptional levels by Polymerase Chain Reaction (PCR). Sequencing results revealed that the full‐length, 3234 bp *GhDSC1 *cDNA (Accession No.:Gh_A10G2076) encodes a protein of 1077 amino acids (aa), and that there is a single intron of 85 bp in the genomic *GhDSC1 *sequence (Fig. S1). Prediction of the protein sequence structure by the web‐based programme SMART showed that *GhDSC1* is a TIR‐NBS‐LRR protein that contains TIR, NBS and LRR domains (Fig. 1A). Phylogenetic analysis with known NBS‐LRR family members showed that CG02 is related to the known resistance gene *DSC1* from *A. thaliana* and *NgN* from *Nicotiana glutinosa*, based on their clustering into an independent branch of the TIR‐NBS‐LRR family (Fig. 1B). Amino acid sequence alignments consisting of those from DSC1 and NgN showed that several distinctive motifs were present in the *GhDSC1‐*encoded protein, including P‐loop, RNBS‐A, Kinase 2, RNBS‐B and RNBS‐C, RNBS‐D and MHD motifs (Figs 1C and S2). Furthermore, analysis of the protein sequence by the web‐based programme LRRfinder (Offord and Werling, 2013) showed that the *GhDSC1* structure includes four typical LRR domains, and two are leucine‐rich‐repeat C‐terminal (LRRCT) domains (Fig. 1C). Together, these results revealed that the cotton Verticillium wilt resistance candidate gene *GhDSC1* encodes a typical TIR‐NBS‐LRR protein structure.

### GhDSC1 localizes to the cell nucleus

**Figure 2:**
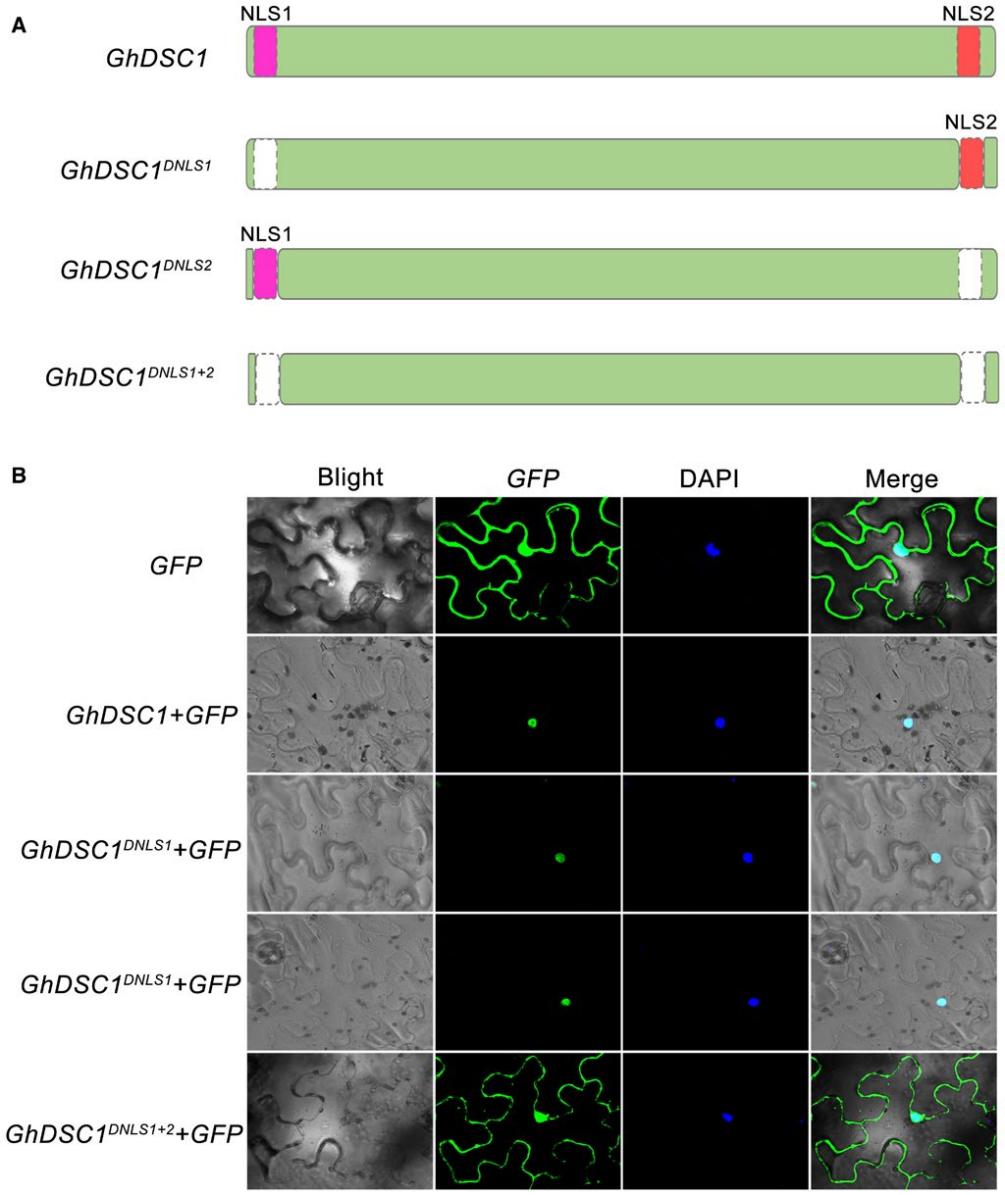
Subcellular localization of *GhDSC1* in *Nicotiana benthamiana*. (A) Structure of nuclear localization signals (NLS) and mutations in *GhDSC1*. NLS1 and NLS2 represent two nuclear localization signals in *GhDSC1*. *GhDSC1^NLS1^*, *GhDSC1^NLS2^*, *GhDSC1^NLS1+2^* represent mutation of NLS1, NLS2 and deletion of NLS1 and NLS2 together, respectively. (B) Subcellular localization of *GhDSC1* and the mutant alleles were determined by transient expression of the C‐terminally green fluorescent protein (*GFP*)‐tagged proteins in *N. benthamiana* leaves. The fluorescence was scanned by a Leica TCS SP8 confocal microscopy system using × 200 magnification with a excitation at 488 nm and emission at 510 nm. The empty vector 35S::*GFP* (*GFP*) was a negative control. Nuclei were stained using 4',6‐diamidino‐2‐phenylindole (DAPI).

To gain insight into the function of *GhDSC1*, the subcellular localization was analysed bioinformatically and experimentally by transient expression of a GhDSC1‐green fluorescent protein (GFP) fusion. Prediction of subcellular localization by the web‐based programme Wolf‐Psort (Horton *et al*., 2007) suggested that GhDSC1 localizes to the cell nucleus (score of nucleus, chloroplast, and plasma is 6, 5 and 1, respectively). According to the prediction information from the web‐based programme cNLS Mapper (Kosugi, *et al*., 2009), GhDSC1 contains two nuclear localization signals (NLS1 and NLS2) (Figs 2A and S3), indicating that GhDSC1 may localize to the cell nucleus. The subcellular location of the GhDSC1‐GFP fusion examined by transient expression in tobacco showed that *GhDSC1* was clearly localized to cell nucleus, in contrast to the fluorescence signal of GFP proteins alone, which was prevalent throughout the foliar cells in tobacco (Fig. 2B). To further confirm the role of NLS1 and NLS2 in localization, individual deletion of NLS1 (*GhDSC1^DNLS1^*) or NLS2 (*GhDSC1^DNLS2^*) and double‐deletion (*GhDSC1^DNLS1+2^*) mutants were constructed for transient expression (Fig. 2A). Interestingly, the effects of the nuclear localization of GhDSC1 could still be observed in individual NLS1 or NLS2 deletion mutants but failed to localize to cell nucleus in NLS1 and NLS2 double‐deletion mutants (Fig. 2B). These results suggested that GhDSC1 is localized to the cell nucleus, and the signal from NLS1 or NLS2 was sufficient for nuclear localization.

### *GhDSC1 *expression is up‐regulated in response to Verticillium wilt and JA signalling in cotton

**Figure 3:**
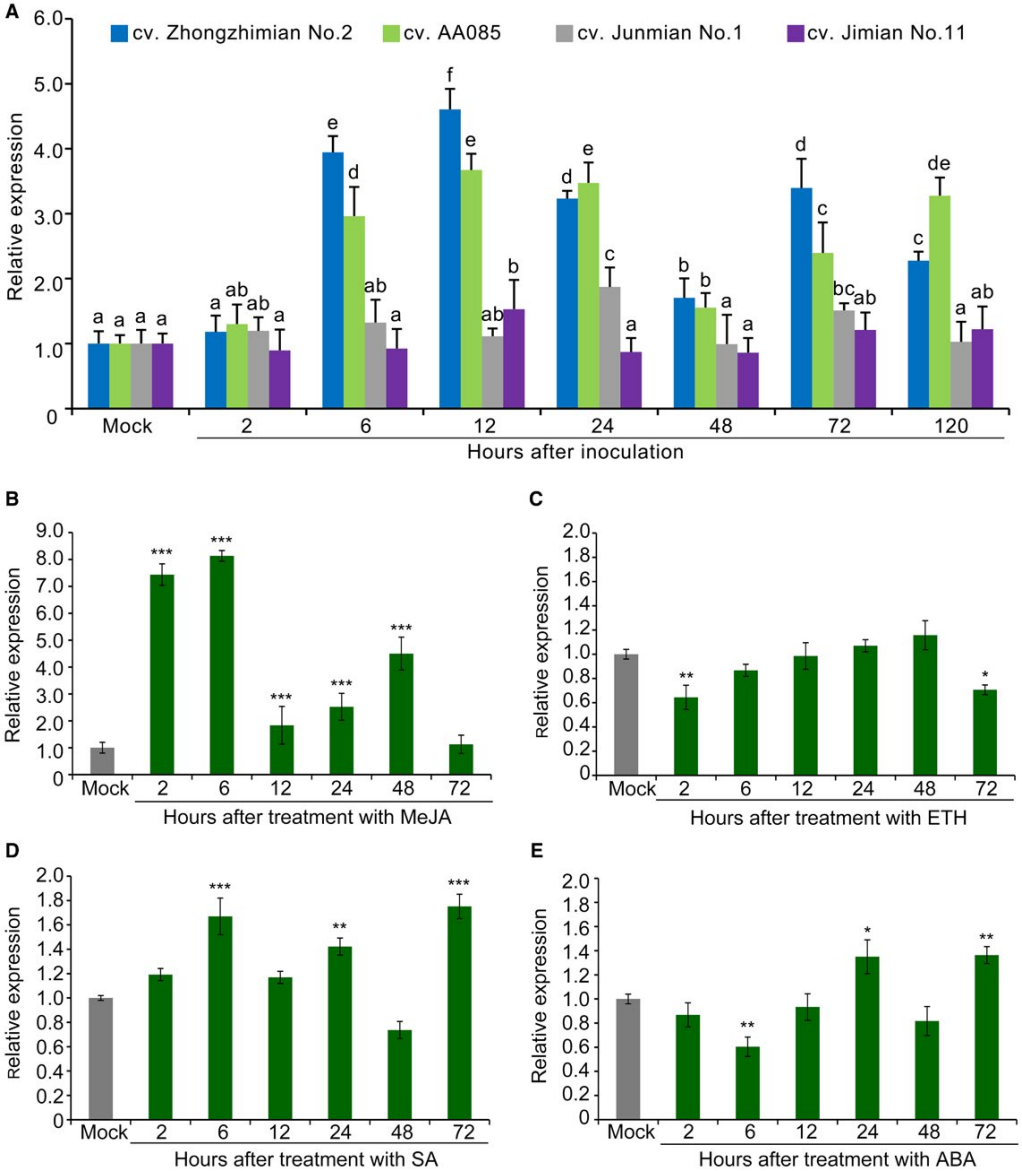
Expression of *GhDSC1 *in response to *Verticillium dahliae* infection and hormone signalling in cotton. (A) Expression analysis of *GhDSC1* in four cotton cultivars over time after inoculation with *V. dahliae *strain Vd991 by Reverse Transcription‐quantitative Polymerase Chain Reaction (RT‐qPCR). Three‐week‐old cotton plants, including two resistance cultivars (cv. Zhongzhimian No. 2 and cv. AA085) and two susceptible cultivars (cv. Junmian No. 1 and cv.Jimian No. 11), were inoculated with conidial suspension (5 × 10^6 ^conidia/mL) and harvested at the respective time points. Different letters indicate significant differences at *P* < 0.01 based on Tukey's HSD. (B–D) *GhDSC1 *expression in response to the four hormone treatments. The transcript levels of *GhDSC1* were detected in 3‐week‐old cotton plants (cv. Zhongzhimian No. 2) treated with the (B) MeJA, (C) ETH, (D) SA and (E) ABA. Relative expression analyses of *GhDSC1* by RT‐qPCR was performed using the cotton *18S* gene as a reference using the comparative threshold 2^‐ΔΔCT^ method, and relative expression was compared with expression levels in cotton plants that were treated with sterile water (Mock). The values shown represent averages of three independent biological replicates of three plants each. Error bars were calculated based on three biological replicates using standard deviation; asterisks (∗) and double asterisks (∗∗) represents statistical significance of *P < *0.05 and *P < *0.01, respectively, according to an unpaired Student's *t*‐tests of each of treatment groups compared with control (Mock).

To test whether *GhDSC1 *expression correlates with Verticillium wilt resistance, expression patterns of *GhDSC1 *in resistant and susceptible cotton cultivars were determined during infection by *V. dahliae*. The expression of *GhDSC1* was significantly up‐regulated in the two resistant cultivars, cv. Zhongzhimian No. 2 and cv. AA085, during the early infection stages (especially 6 h–24 h after inoculation) (Fig. 3A). Conversely, in the susceptible cotton cv. Junmian No. 1 and cv. Jimian No. 11, the transcript levels of *GhDSC1* did not significantly change until 120 h after inoculation with *V. dahliae* (Fig. 3A), suggesting that the expression of *GhDSC1* positively correlated with Verticillium wilt resistance in cotton. The involvement of *GhDSC1 *in Verticillium wilt resistance was also evident in the expression pattern of *GhDSC1* in different cotton tissues at the adult‐plant stage, which was significantly up‐regulated in root, stem and petiole tissues compared with the expression in leaf at 72 h after flooding with *V. dahliae* conidial suspension (Fig. S4). To identify signalling pathway(s) linked with *GhDSC1*, the expression pattern of *GhDSC1* was examined following treatment with salicylic acid (SA), ethephon (ETH), methyljasmonate (MeJA) and abscisic acid (ABA), respectively. Interestingly, the expression pattern of *GhDSC1 *was affected after application of MeJA, but not SA, ETH or ABA (Fig. 3B,C,D,E). These results suggested that *GhDSC1 *expression is mediated by JA signalling.

### GhDSC1 enhances resistance to Verticillium wilt in *Arabidopsis thaliana*

**Figure 4:**
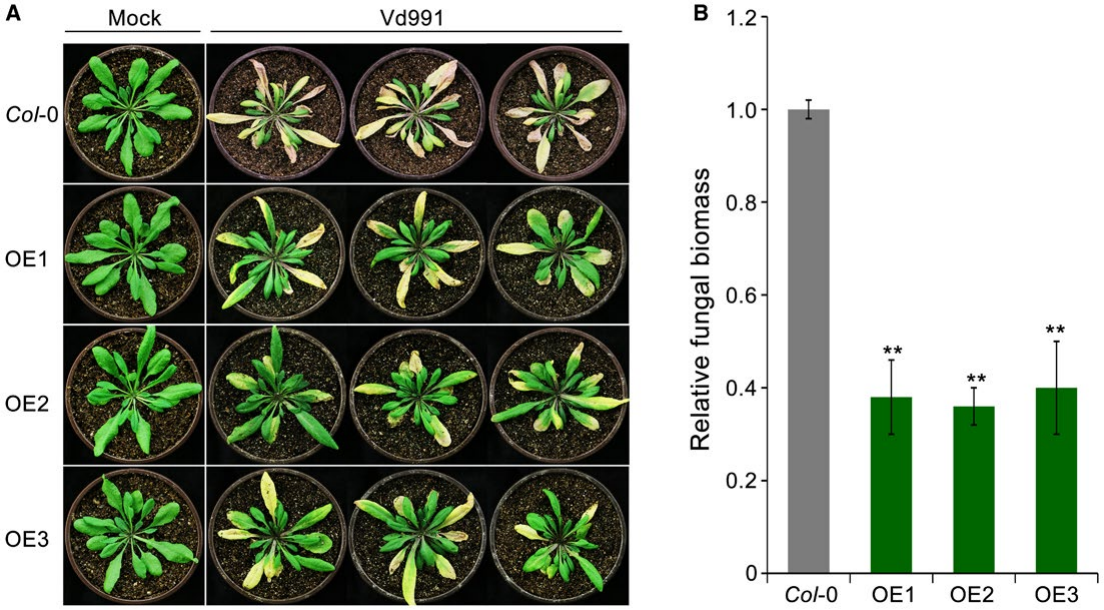
Transgenic expression of *GhDSC1* enhances Verticillium wilt resistance in *Arabidopsis thaliana*. (A) Identification of Verticillium wilt resistance after interfamily transfer of *GhDSC1* in *A. thaliana*. Three‐week‐old seedlings of homozygote transgenic *A. thaliana* (T_3_) were inoculated with 5 mL of *Verticillium dahliae* conidial suspension (5 × 10^6 ^conidia/mL). The Verticillium wilt phenotypes were determined and photographed 3 weeks after inoculation. Mock, inoculation with sterile water; OE1/OE2/OE3, *GhDSC1 *overexpressing transgenic plants. (B) Quantification of *V. dahliae* biomass in *GhDSC1* transgenic *A. thaliana* plants (OE1, OE2 and OE3) compared to the wild‐type (Col‐0). Genomic DNA was extracted from three whole plants at 21 days after inoculation, and the relative fungal biomass was determined using quantitative Polymerase Chain Reaction (qPCR). *V. dahliae* elongation factor 1‐α (*EF‐1α*) was used to quantify fungal colonization, and *A. thaliana UBQ5 *was used as endogenous plant control. Error bars represent standard errors of three biological replicates, asterisks (∗∗) indicates statistical significance (*P < *0.01), according to unpaired Student's *t*‐tests of plants of each OE compared to the wild‐type (Col‐0).

To investigate the role of *GhDSC1* in the defence against *V. dahliae*, *GhDSC1 *was heterologously expressed in *A. thaliana*. The *GhDSC1* expression construct, in which *GhDSC1* expression was driven by the CaMV35S (35S) promoter (P35S::*GhDSC1*), was transferred into *A. thaliana* (ecotype Col‐0) via *Agrobacterium tumefaciens*‐mediated transformation. Positive transgenic lines were verified by PCR and the expression of *GhDSC1* was confirmed by Reverse Transcription (RT)‐PCR (Fig. S5A). Six independent *GhDSC1*‐transgenic lines (T_3 _generation) were obtained (Fig. S5B). Verticillium wilt resistance was evaluated using the highly virulent *V. dahliae* strain Vd991 on 4‐week‐old seedlings of three OE transgenic lines (OE1–OE3) that were arbitrarily selected. The results showed that the *GhDSC1*‐overexpressing lines exhibited significantly enhanced resistance to *V. dahliae* Vd991, as indicated by reductions in leaf chlorosis and withering compared to the wild‐type Col‐0 (Fig. 4A). Furthermore, real‐time quantitative PCR (qPCR) demonstrated that the *GhDSC1*‐transgenic lines developed significantly less fungal biomass *in planta* than the wild‐type *A. thaliana* plants (Fig. 4B). Thus, *GhDSC1 *conferred resistance to *V. dahliae* even after the interfamily transfer into *A. thaliana*.

### *GhDSC1 *can restore Verticillium wilt resistance in the *Arabidopsis thaliana dsc1 *mutant background

**Figure 5:**
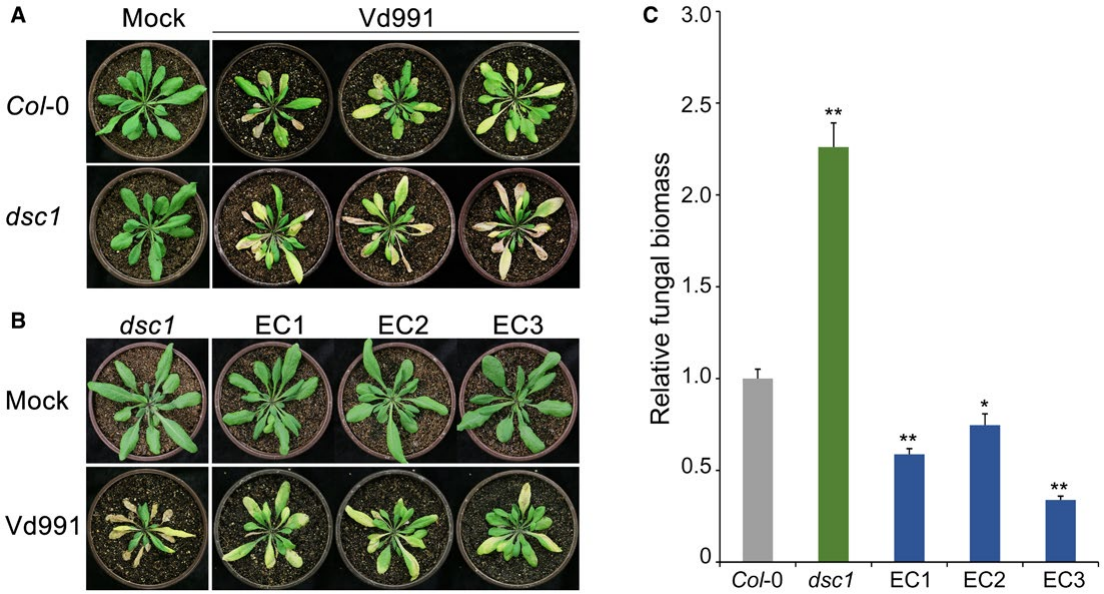
*GhDSC1 *complements Verticillium wilt resistance in an *Arabidopsis thaliana dsc1 *mutant*. *(A) Verticillium wilt phenotype *A. thaliana* line *dsc1*, a T‐DNA mutant of *DSC1, *which is orthologous to *GhDSC1*. (B) Identification of Verticillium wilt resistance of ectopic transformants in which *GhDSC1* was introduced into the *dsc1 *mutant. Three‐week‐old transgenic (complement transformants) lines (EC1, EC2 and EC3) were subjected to a root‐dip inoculation in a suspension of 5 × 10^6 ^conidia/mL of *Verticillium dahliae*, strain Vd991. Verticillium wilt symptoms were assessed 21 days after inoculation. Sterile water was used in controls (Mock). (C) Quantification of *V. dahliae* biomass in transgenic *GhDSC1* lines of the *dsc1 *mutants by quantitative Polymerase Chain Reaction (qPCR). Error bars represent standard errors of three biological replicates, asterisks (∗) and double asterisks (∗∗) represents statistical significance of *P* < 0.05 and *P* < 0.01, respectively, according to unpaired Student's *t*‐tests between *dsc1 *mutants and EC plants compared with the wild‐type (Col‐0).

The *A. thaliana DSC1 *is the orthologue of *GhDSC1* (Gh_A10G2076) in *G. hirsutum *(Zhang *et al*., 2015). BLASTp analysis using GhDSC1 as a query against *A. thaliana* proteins returned DSC1 (AT4G12010.1) as the best hit (amino acid identities = 354/1167, 30%; positives = 560/1167, 47%), which also has the typical TIR‐NBS‐LRR motif (Fig. S2), and the closest phylogenetic relationship (Fig. 1B). Because we had identified a role for *GhDSC1* in Verticillium wilt resistance, we hypothesized that its orthologue, *DSC1*, also confers Verticillium wilt resistance in *A. thaliana*. To test this hypothesis, the sensitivity of *DSC1 *homozygosis mutant (*dsc1*, Stock ID in TAIR:SALK_014299) to *V. dahliae* was examined using the root‐dip inoculation method. The results showed that the *dsc1 *mutant grew normally as the wild‐type Col‐0 ecotype after disruption of *DSC1* in *A. thaliana* but was more sensitive to *V. dahliae* compared with the wild‐type Col‐0 ecotype, showing significant leaf chlorosis and wilting 2 weeks after inoculation (Fig. 5A). Investigation of the fungal biomass by qPCR revealed rapid *V. dahliae *multiplication in the *dsc1* lines relative to the wild‐type Col‐0 ecotype (Fig. 5C). These results suggested that the orthologue gene *DSC1* is also involved in Verticillium wilt resistance in *A. thaliana*. To further confirm that the orthologue gene *DSC1 *is involved in Verticillium wilt resistance, the sensitivity to *V. dahliae* was assessed in the mutant *dsc1* of *A. thaliana* following the complementation of *GhDSC1 *driven by the 35S promoter. As expected, inoculation of three separate *dsc1* transgenic lines complemented with *GhDSC1 *displayed significantly less chlorosis and wilting compared with the *dsc1* mutants (Fig. 5B). Correspondingly, the fungal biomass was significantly less in the *GhDSC1*‐recepient *dsc1* mutants (Fig. 5C). Therefore, our results showed that both *GhDSC1* and its orthologous gene (*DSC1*) share a common function in contributing to Verticillium wilt resistance in *A. thaliana*. The heterologously expressed *GhDSC1* could also compensate for the Verticillium wilt sensitivity in the *A. thaliana* *dsc1* mutant, providing confirmation that *GhDSC1* confers Verticillium wilt resistance.

### GhDSC1 modulates defence responses of ROS accumulation and expression of JA‐regulated defence genes

**Figure 6:**
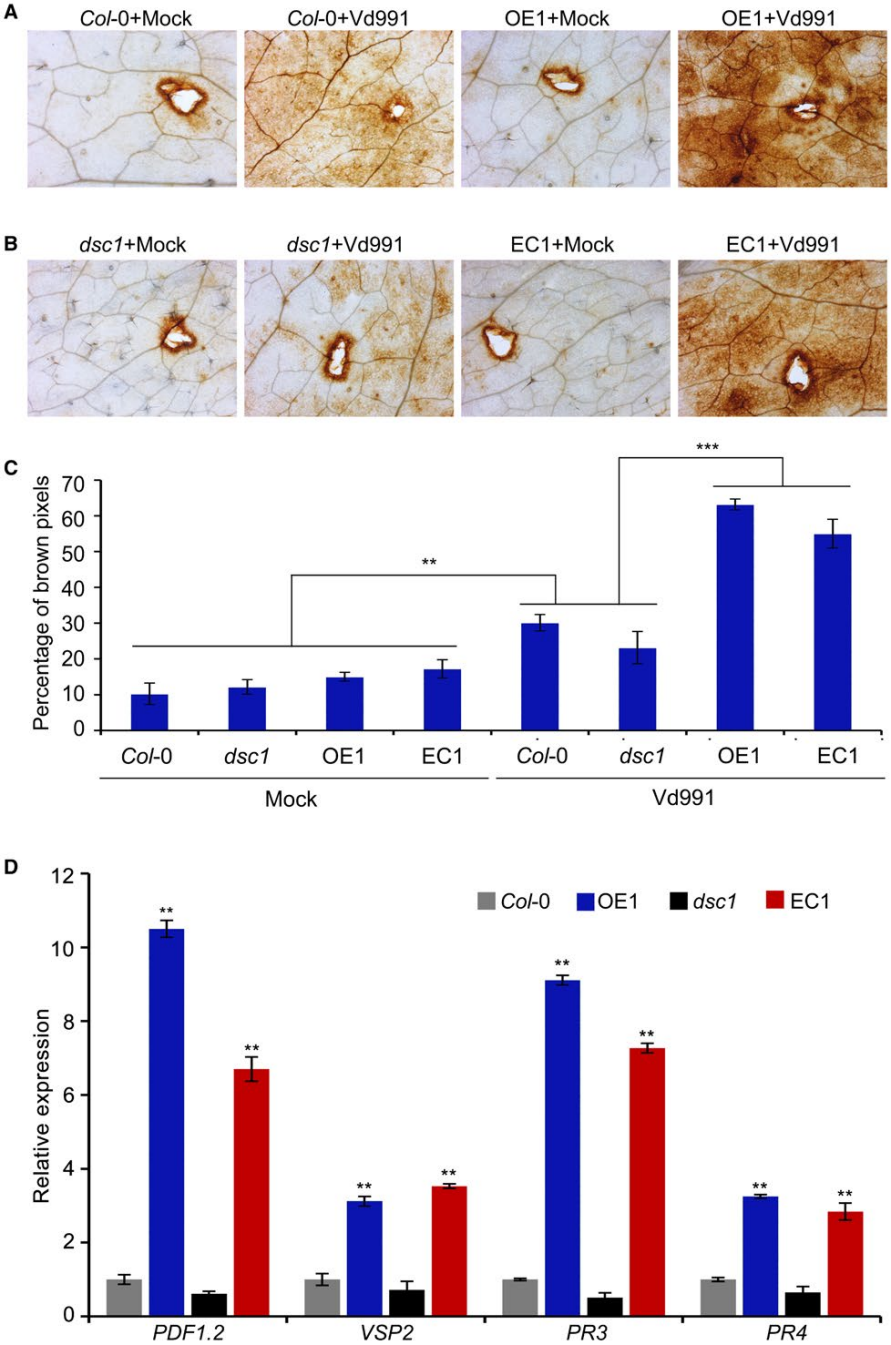
Identification of *GhDSC1‐*mediated defence responses in *Arabidopsis thaliana*. (A) Diaminobenzidine staining of ROS accumulation in *A. thaliana *transgenic line that overexpressed *GhDSC1*. ROS accumulation was assessed in *GhDSC1* transgenic *A. thaliana* and wide type (Col‐0) leaves from 3‐week‐old plants 12 h after infiltration with a 10 μL suspension (5 × 10^6^ conidia/mL) of *Verticillium dahliae* strain Vd991. Sterile water treatment was used as a control (Mock). ROS accumulation was captured by the microscopy with 13.5 × amplification under the stereomicroscope. (B) Detection of ROS‐inducing activities of *A. thaliana dsc1 *mutants and *dsc1* mutants after introduction of *GhDSC1*. (C) The percentages of brown pixels of transgenic plants inoculated with *V. dahliae* strain Vd991. These included the *GhDSC1 *overexpression transgenic *A. thaliana* (OE1), *A. thaliana dsc1* mutant that received *GhDSC1* (EC1), wild‐type (Col‐0) and the *A. thaliana dsc1* mutant. Values are means ± SD from three independent experiments. Asterisks (**) and (***) indicate a significant difference (*P < *0.01) and (*P < *0.005) relative to the control with sterile water (Mock) based on unpaired Student's *t*‐test. (D) Identification of the JA signalling‐associated gene expression mediated by *GhDSC1* in *A. thaliana*. Overexpression transgenic line (OE1), *dsc1 *mutant, transgenic lines of the *dsc1* mutant that introduced *GhDSC1* (EC1), and wild‐type (Col‐0) were inoculated with a conidial suspension of 5 × 10^6 ^conidia/mL of *V. dahliae* strain Vd991 using a root‐dip method. Leaf samples were collected 24 h after inoculation. Relative expression was assessed by Reverse Transcription‐quantitative Polymerase Chain Reaction (RT‐qPCR) using the comparative threshold 2^‐∆∆CT^ method and *A. thaliana UBQ5 *as a reference. Values represent averages of three independent biological replicates. Error bars represent standard errors. Double asterisks (∗∗) represent statistical significance of *P < *0.01, according to an unpaired Student's *t*‐tests of each *dsc1 *mutant, EC1 and OE1 plants compared with the wild‐type (Col‐0).

To explore the potential mechanisms of *GhDSC1*‐mediated Verticillium wilt resistance in *A. thaliana*, ROS accumulation was assessed in *GhDSC1*‐transgenic lines during *V. dahliae* infection. ROS accumulation was assessed in leaves of *A. thaliana *ecotype Col‐0, transgenic lines overexpressing *GhDSC1*, *dsc1* mutants and in the *GhDSC1*‐receipient *dsc1* mutants following infiltration of a conidial suspension of *V. dahliae* strain Vd991. Leaves of both the wild‐type Col‐0 and the *GhDSC1*‐transgenic lines registered an enhanced ROS accumulation around the infiltration sites (indicated by dark brown deposits visible in leaves) 12 h after conidial infiltration, compared with leaves infiltrated with sterile water (Fig. 6A,C). In contrast to the wild type, however, the ROS accumulation in the *GhDSC1*‐transgenic lines was significantly enhanced 12 h after inoculation with *V. dahliae* (Fig. 6A). Furthermore, the *dsc1* mutants displayed relatively lower ROS accumulation compared to the wild‐type Col‐0 because of the disruption of *DSC1* in *A. thaliana* (Fig. 6A,B). Again, ROS was significantly up‐regulated 12 h after *V. dahliae *inoculation in the *GhDSC1*‐recipient *dsc1* mutant (Fig. 6B,C). Expression analysis showed that the transcript levels of *GhDSC1* were similar in the recipient Col‐0 and *dsc1* mutant, which corresponded to the similar ROS accumulation between transgenic lines overexpressing *GhDSC1* and the *GhDSC1*‐receipient *dsc1* mutants (Figs 6C and S6). These results suggested that *GhDSC1* activates ROS accumulation to enhance Verticillium wilt resistance.

The expression patterns of *GhDSC1* in cotton (Fig. 3A) suggested the involvement of JA signalling. To test this hypothesis, the relative expression of four JA pathway‐regulated genes (*PDF1.2*, *VSP2*, *PR3* and *PR4*) were examined in the background of *A. thaliana* plants in which *GhDSC1 *was absent or overexpressed. Compared to the transcript levels observed in the wild‐type Col‐0, all of the JA‐regulated genes were significantly up‐regulated in transgenic lines overexpressing *GhDSC1*. Additionally, these genes were significantly down‐regulated in the *dsc1 *mutant (Fig. 6D). Therefore, in addition to the activation of ROS accumulation, these results indicated that *GhDSC1* activates defence responses through JA signalling to confer Verticillium wilt resistance.

### Similar expression patterns of *GhCAMTA3 *and *GhDSC1 *in response to Verticillium wilt and JA signalling in cotton

**Figure 7:**
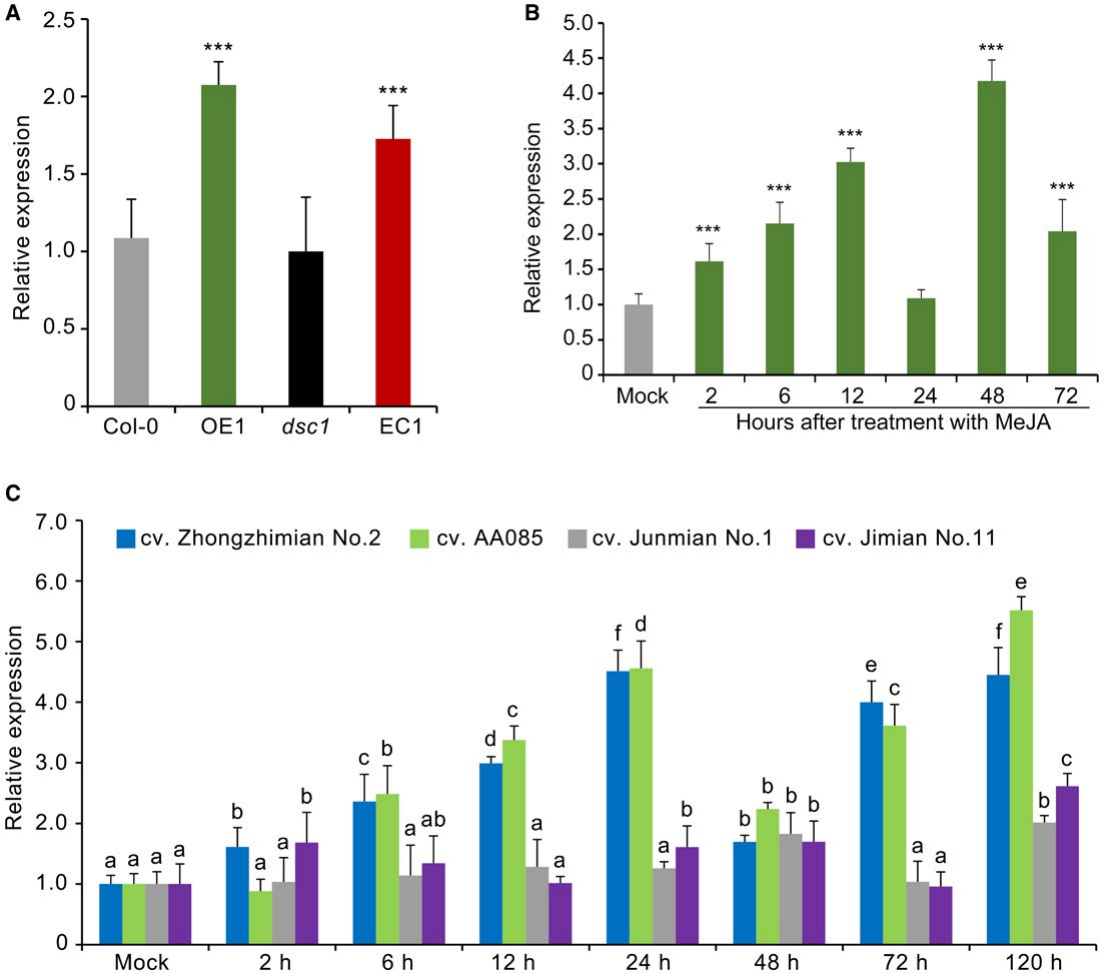
*GhDSC1 *and *GhCAMTA3* show similar expression patterns in cotton. (A) Expression analysis of *GhCAMTA3* in the *GhDSC1* overexpression transgenic line (OE1), the *dsc1 *mutant, transgenic lines of *dsc1* mutant in which *GhDSC1* was introduced (EC1), and wild‐type Col‐0. The respective plants were inoculated with a conidial suspension of 5 × 10^6^ conidia/mL of *Verticillium dahliae* (strain Vd991) using a root‐dip method. Leaf samples were collected 72 h after inoculation. The transcript relative expression was assessed by Reverse Transcription‐quantitative Polymerase Chain Reaction (RT‐qPCR) using the comparative threshold 2^‐∆∆CT^ method and the *Arabidopsis thaliana UBQ5* as a reference. Values represent averages of three independent biological replicates. Error bars represent standard errors. Double asterisks (∗∗) represent statistical significance of *P < *0.01, according to an unpaired Student's *t*‐tests between *dsc1 *mutants, EC1 and OE1 plants compared with the wild‐type (Col‐0). (B) Expression of *GhCAMTA3* in response to MeJA treatment. Transcript levels of *GhCAMTA3* were detected in RNA samples from 3‐week‐old cotton plants (cv. Zhongzhimian No. 2) treated with 10 mM MeJA. Asterisks (∗∗) and (***) represent statistical significance at *P < *0.01 and *P < *0.005, respectively, according to unpaired Student's *t*‐tests between treatment groups compared with the control group (Mock). (C) Expression analysis of *GhCAMTA3* in four cotton cultivars after inoculation with *V. dahliae *strain Vd991 by RT‐qPCR. The samples of four cotton cultivars were treated as in detection of the expression of *GhDSC1*. Relative expression analyses of *GhDSC1* by RT‐qPCR were performed using the cotton *18S* gene as reference using the comparative threshold 2^‐ΔΔCT^ method. Values represent the averages of three independent biological replicates of three plants each. Error bars represent standard errors. Different letters indicate significant differences at *P* < 0.01 based on Tukey's HSD.

Previous studies had shown that *DSC1* functions in part through its association with the Calmodulin Binding Transcription Activator 3 (*CAMTA3*), which acts as a negative regulator of immunity to inhibit *DCS1‐*induced autoimmunity (Lolle *et al*., 2017)*. *Comparative genomics revealed that one gene encodes *CAMTA3* in the cotton (*G. hirsutum*) genome (hereafter referred to as *GhCAMTA3*) (Zhang *et al*., 2015). The expression of *GhCAMTA3*, as affected by *GhDSC1*, was detected in *A. thaliana* transgenic lines. The results showed that the transcript levels of *GhCAMTA3* were significantly up‐regulated in the *GhDSC1*‐transgenic line compared with the wild‐type Col‐0, and similar results were recorded in the *dsc1* mutant and the *GhDSC1*‐recipient *dsc1 *transgenic line, (Fig. 7A). To further explore the relationship between the expression patterns of *GhCAMTA3* and *GhDSC1*, the transcript levels of *GhCAMTA3* and *GhDSC1 *were detected following the same treatments. The expression levels of both *GhCAMTA3* and *GhDSC1* were also significantly enhanced after application of the MeJA at 6 h to 72 h (Fig. 7B), but not following treatments with ETH, SA and ABA (Fig. S7). Similar to the increased expression of *GhDSC1* observed following *V. dahliae* inoculation, the expression of *GhCAMTA3* in resistant plants (cv. AA085 and cv. Zhongzhimian No. 2) was induced 2 h–120 h following *V. dahliae *inoculation, when compared with the susceptible plants (cv. Junmian No. 1 and cv. Jimian No. 11) (Fig. 7C). The expression pattern of *GhCAMTA3* was strikingly similar to patterns observed for *GhDSC1*, in response to multiple treatments that stimulate plant defence.

### A nonsynonymous mutation in the P‐loop motif of GhDSC1 differentiates resistance and susceptible cotton cultivars

**Figure 8:**
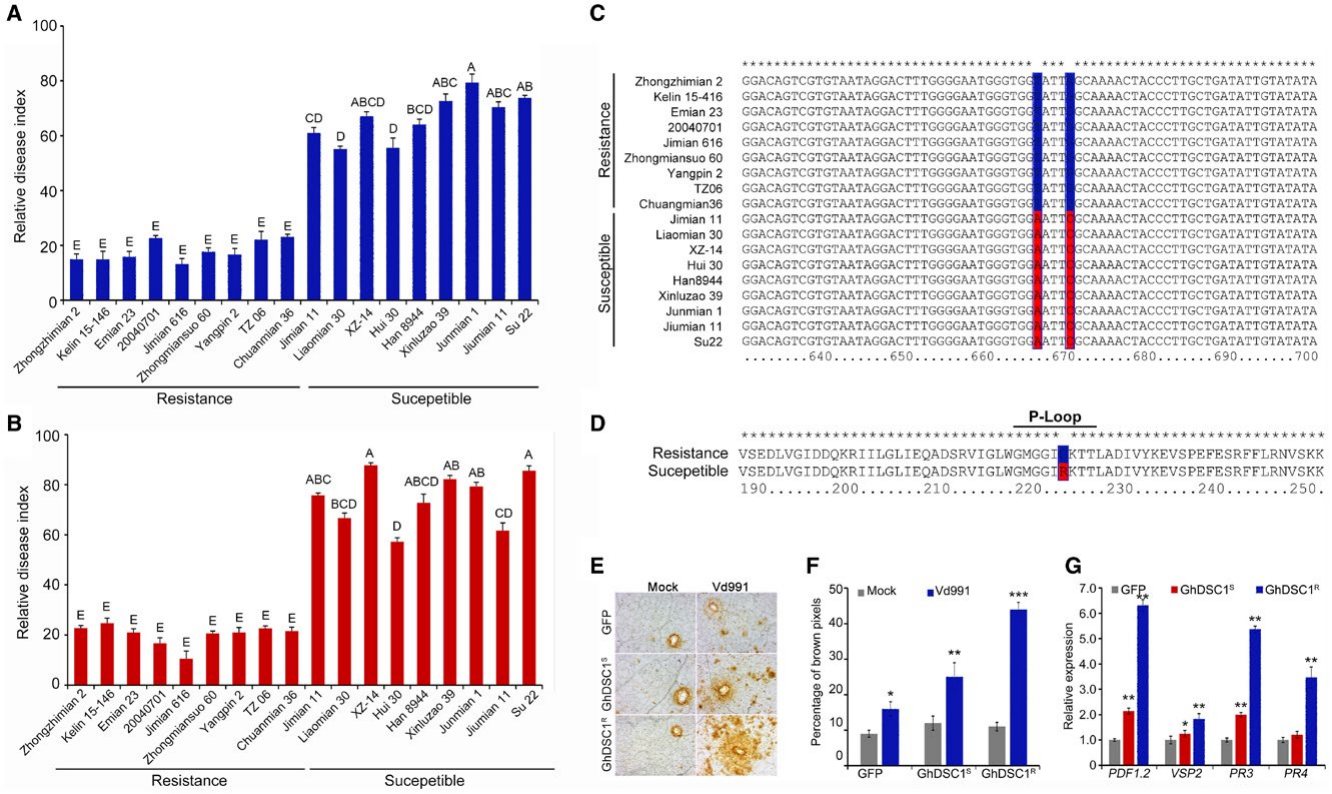
*GhDSC1 *sequence divergence in resistant and susceptible cotton cultivars and introduction of a P‐loop mutation. (A and B) Relative disease index of the *Gossypium hirsutum *germplasm accessions in response to *Verticillium dahliae*. Eighteen cotton germplasm accessions (nine are resistance phenotype) were selected for sequence divergence analysis; the relative disease index of Verticillium wilt was deployed previously (Li et al., 2017), and investigated in field (A) and greenhouse (B) experiments by a root‐dip inoculation method. Bars in green and red colour represent the relative disease index in the field and greenhouse, respectively. Error bars represent standard errors in a sample of three plants. Different letters indicate significant differences at *P* < 0.01 as calculated by one‐way analysis of variance (ANOVA) with Tukey's HSD used for mean comparisons.(C) Partial alignment of *GhDSC1 *homologues isolated from all *G. hirsutum *germplasm accessions. The partial alignment represents the only nonsynonymous mutation site (673 bp, GGC >> CGC) in *GhDSC1 *amongst *G. hirsutum *germplasm accessions, the resistant and susceptible nucleotide bases were labelled in green and red colour, respectively. Asterisks (∗) represent nucleotide base conservation. (D) Amino acid alignment of *GhDSC1* between resistant and susceptible cotton cultivars. The genotype of *GhDSC1* has a nonsynonymous mutation (225 aa, G >> R) in the P‐loop motif, and the amino acid residues of resistant and susceptible cultivars were marked in green and red colour, respectively. (E) Comparison of the ROS accumulation by transient expression between the resistant genotype *GhDSC1^R^* and susceptible genotype *GhDSC1^S^*. *Nicotiana benthamiana* leaves from 4‐week‐old plants were agroinfiltrated with *GhDSC1^R^* and *GhDSC1^S^*, respectively. Two days later, plants were inoculated with a conidial suspension of 5 × 10^6 ^conidia/mL of *V. dahliae* or sterile water (Mock). ROS accumulation stained with DAB solution was detected 2 days after inoculation. Agro‐infiltration of *GFP* served as a control. (F) The percentages of brown pixels of *N. benthamiana *after introduction of *GFP, GhDSC1^R^* and *GhDSC1^S^*. Asterisks (∗), (∗∗) and (∗∗∗) represents statistical significance at *P < *0.05, *P < *0.01 and *P < *0.005 according to unpaired Student's *t*‐tests of each of the treatment groups compared to the control group (Mock). (G) Expression of JA signalling‐associated genes 2 days after transient expression of *GFP, GhDSC1^R^* and *GhDSC1^S^* in *N. benthamiana*. Asterisks (∗) and double asterisks (∗∗) represent statistical significance at *P < *0.05 and *P < *0.01, respectively, according to unpaired Student's *t*‐tests of each of the treatment groups compared to the control group.

A GWAS revealed that *GhDSC1* (CG02) was located in the Verticillium wilt resistance locus in cotton (Li *et al*., 2017), suggesting that *GhDSC1* may be conserved amongst the resistant cotton germplasm. To explore the genetic divergence of *GhDSC1* in resistant and susceptible cultivars, nine typical resistant and susceptible *G. hirsutum *germplasm accessions were selected for sequence analyses (Table S1). The Verticillium wilt resistant germplasm accessions were analysed by both a disease index in a diseased field nursery and in a greenhouse after inoculations with *V. dahliae*. The average disease indices of the resistant germplasm was 20 compared with 60 for the susceptible germplasm (Fig. 8A,B). Furthermore, open reading frames (ORF) of *GhDSC1* homologues were PCR‐amplified and sequenced from all 18 cotton germplasm accessions. Alignments of the ORF sequences showed that the *GhDSC1* homologues were highly conserved amongst the 18 cotton accessions, except for 11 single nucleotide polymorphisms (SNPs) (Fig. S8). Of these SNPs, the polymorphism at 673 bp was a guanine (G) in resistant accessions but was cytosine (C) in susceptible accessions (Fig. 8C). This change represented the one nonsynonymous SNP (GGC >> CGC) that resulted in amino acid sequence divergence (225 aa, G >> R) (Fig. 8D). Interestingly, sequence analyses further revealed that this nonsynonymous mutation occurred in the P‐loop motif (Fig. 8D). The P‐loop motif plays a critical role in the functioning of TIR‐NBS‐LRR proteins (Hishida *et al*., 1999; Traut, 1994). These results indicated that Verticillium wilt resistance function of *GhDSC1* may be distinguished by the nonsynonymous mutation between the resistant and susceptible cotton *GhDSC1 *sequences.

To determine the functional divergence of *GhDSC1*, differences in defence response activation were analysed following transient expression of the *GhDSC1* resistant genotype (*GhDSC1^R^*) and the susceptible genotype (*GhDSC1^S^*) and in *Nicotiana benthamiana*. ROS accumulation was similar 2 days after transient expression of *GhDSC1^R^* and *GhDSC1^S^* following treatment with sterile water but was significantly higher following inoculations with *V. dahliae *in the *GhDSC1^R^* compared to the *GhDSC1^S ^*plants (Fig. 8E,F). The transcript levels of four JA‐regulated genes (*PDF1.2*, *VSP2*, *PR3* and *PR4*) were significantly up‐regulated after transient expression of *GhDSC1^R^* compared to the *GhDSC1^S ^*(Fig. 8G), further suggesting that the GhDSC1 may efficiently activate the defence response. These results suggested that *GhDSC1* is associated with the Verticillium wilt resistance in cotton, and that the sequence divergence causing the P‐loop mutation determines the resistance or susceptibility of cotton to Verticillium wilt.

## Discussion

Several genes in cotton that contribute to defence responses against Verticillium wilt have been characterized (Duan *et al*., 2016; Gao *et al*., 2013; Li *et al*., 2016, 2018c, 2016, 2018c; Mo *et al*., 2015; Munis *et al*., 2010; Yang *et al*., 2015, 2016, 2015, 2016; Zhang *et al*., 2011; 2012; 2013b; 2015), and studies on these genes provide an ever greater understanding of the bases for disease resistance in cotton. In most of these studies, however, candidate genes were identified in an arbitrary model such as conserved homologue cloning that lacks a genetic basis. Genetic methods to identify those genes that play a role in resistance are effective and practical, and also valuable for improved genetic selection by molecular breeding, but the complexity of allotetraploid genome has hitherto prevented such studies in cotton. In our previous study (Li *et al*., 2017), a GWAS was performed using a population of 299 cotton (*G. hirsutum*) germplasm accessions and a Verticillium wilt candidate resistance gene, *GhDSC1*, was identified from the associated locus. In this study, the role of *GhDSC1* in Verticillium wilt resistance was examined from several different angles, including its expression and localization in cotton and its heterologous expression and phenotypic characterization representing resistant and susceptible responses in *A. thaliana*. We also observed that ROS activation and JA signalling were associated with *GhDSC1*‐mediated Verticillium wilt resistance in cotton.

Most of the disease resistance genes characterized in plants that encode NBS‐LRR proteins play key roles in pathogen resistance by activating defence responses (DeYoung and Innes, 2006). Generally, NBS‐LRR proteins are involved in the recognition of specialized pathogen effectors to activate the innate immunity (defence response) against pathogen invasion in two main mechanisms: detection through direct interaction of plant NBS‐LRR proteins and pathogen‐derived molecules or detection indirectly through the action of their effectors (guard model), which allows the plant to monitor a limited number of key targets of pathogenesis, and responds when those targets are perturbed (Chisholm *et al*., 2006; Van der Biezen and Jones, 1998; DeYoung and Innes, 2006). The NBS‐LRR family is encoded by hundreds of diverse genes per genome and can be subdivided into two functionally distinct subfamilies of TNL and CNL proteins (McHale *et al*., 2006), and many of these contribute to resistance (Belkhadir *et al*., 2004; Joshi and Nayak, 2011). In cotton, the genome encodes a large number of NBS‐LRR proteins (Chen *et al*., 2015; Khan *et al*., 2016), which have been a focus of attention for their respective functions in disease resistance, especially for Verticillium wilt.

Comparative genomic analysis showed that the expansion and contraction in the numbers of NBS‐encoding genes in different cotton species alter their resistance to *V. dahliae* (Li *et al*., 2014), and many of them are also involved in host responses during infection (Chen *et al*., 2015; Li *et al*., 2014; Xu *et al*., 2011b; Zhang *et al*., 2013b). However, except for *GbRVd* and *GbaNA1* (Li *et al*., 2018a; Yang *et al*., 2016), few have been definitively characterized as contributing to Verticillium wilt resistance. We had previously identified *GhDSC1 *(typical TIR‐NBS‐LRR) (Fig. 1) by mining the GWAS using a population of *G. hirsutum* accessions (Li *et al*., 2017). Following overexpression in *A. thaliana*, *GhDSC1* conferred Verticillium wilt resistance, and also restored resistance in the *A. thaliana* *dsc1 *mutant (Figs 4 and 5). This is perhaps the first NBS‐LRR gene associated with Verticillium wilt resistance that was identified and cloned using the genetic screen employed. This demonstrates the utility of such an approach to uncover mechanisms of Verticillium wilt resistance and augment molecular breeding strategies.

Plants have developed complex defence systems against diverse pathogens, systems, which comprise various responses to prevent infection (Caplan *et al*., 2008; DeYoung and Innes, 2006; Elmore *et al*., 2011; van Loon *et al*., 2006). In cotton, the chief defence mechanisms depend on pre‐formed defence structures including a thick cuticle, synthesis of phenolic compounds and delaying the invader through reinforcement of cell walls, accumulation of ROS, and release of phytoalexins (Shaban *et al*., 2018). For instance, a thioredoxin (GbNRX1) that scavenges apoplastic ROS following the ROS burst upon recognition of *V. dahliae* is critical for the apoplastic immune response (Li *et al*., 2016). Similarly, the defence responses mediated by overexpressed *GhDSC1* also resulted in the ROS accumulation in *A. thaliana *(Fig. 6A,C) and following complementation with *GhDSC1* in the *A. thaliana dsc1* mutant (Fig. 6B,C). ROS accumulation as a Verticillium wilt resistance response also has been reported in association with several other candidate genes, including *GbaNA1* (Li *et al*., 2018a), *Gh‐LYK1* and *Gh‐LYK2* (Gu *et al*., 2017) and *GbRVd* (Yang *et al*., 2016), suggesting that ROS accumulation plays a critical role in cotton resistance to *V. dahliae*. Furthermore, hormone‐mediated signalling is one of the most important aspects of this defence mechanism, (Fujita *et al*., 2006), and SA, JA and ETH are three main hormones contributing to defence against *V. dahliae* (Duan *et al*., 2016; Gao *et al*., 2013; Guo *et al*., 2016; He *et al*., 2017; Li *et al*., 2014, 2018b, 2014, 2018b; Mo *et al*., 2015; Parkhi *et al*., 2010; Sun *et al*., 2014; Wang *et al*., 2017; Xu *et al*., 2014; Yang *et al*., 2015; Zhang *et al*., 2013b; Zuo *et al*., 2007). Several functional studies have explored the roles of novel genes implicated in cotton defence, and some of these modulate JA signalling, such as *GbSBT1* (Duan *et al*., 2016), *GbSSN* (Sun *et al*., 2014), *GhNINJA* (Wang *et al*., 2017), *GhJAZ2 *and *GhbHLH171* (He *et al*., 2017) and *GbSSI1* (Gao *et al*., 2013). In this study, we found that the expression of *GhDSC1 *was significantly up‐regulated after treatment with MeJA (Fig. 3B), and marker genes of JA signalling displayed a positive correlation with the presence of *GhDSC1* (transgenic lines) (Fig. 6D). Interestingly, the expression levels of *GhDSC1 *were not altered following treatment with ETH (Fig. 3C), but ETH and JA are usually considered to act synergistically against *V. dahliae* (Xu *et al*., 2011a). Thus, these results indicated that *GhDSC1‐*associated defence responses to Verticillium wilt are mediated via JA signalling in cotton.

Analysis of the characteristic structural features within the translated *GhDSC1* sequence revealed characteristics of a typical TIR‐NBS‐LRR protein, orthologous to DSC1 in *A. thaliana*. The *A. thaliana* *DSC1* encodes a typical TIR‐NBS‐LRR, part of an NLR pair with the TIR‐NBS‐LRR At4g12020 (*DSC2*), similar to the *RPS4* and *RRS1* pair in *A. thaliana* (Narusaka *et al*., 2009). Interestingly, the *GhDSC1* locus encodes another typical TIR‐NBS‐LRR protein (Gh_A10G2077, CG03) that also appears to be a part of an NLR head‐to‐head pair together with GhDSC1 (Li *et al*., 2017). In *A. thaliana*, the expression analysis of the majority of NBS‐LRR‐encoding genes showed that DSC1 is affected by SA or flg22 (Meyers *et al.,* 2003). DSC1 is responsible for immunity in *N. benthamiana *since *Agrobacterium *expressing *DSC1* resulted in HR, and the immunity can be suppressed by CAMTA3 as demonstrated by the co‐inoculation with *CAMTA3* that inhibited the *DSC1*‐induced cell death. This suggests that *DSC1* and *CAMTA3* represent a guard/guardee pair as proposed by Lolle *et al*. (2017). Interestingly, the expression patterns of *GhCAMTA3* (Gh_D12G0791, *CAMTA3* orthologue gene in cotton) and *GhDSC1 *displayed similarities after inoculation with *V. dahliae* or treatment with JA (Fig. 7A,B), and the transcript levels of *DSC1* were also enhanced after overexpressing *GhDSC1* in wild‐type Col‐0 and the *dsc1* mutant (Fig. 7B). *GhDSC1* was further localized to the cell nucleus, corresponding to the findings of the localization of CAMTA3 and DSC1 that interact together in the cell nucleus (Lolle *et al*., 2017). However, GhDSC1 and GhCAMTA3 did not display interaction in a yeast two‐hybrid analysis (Fig. S9), suggesting that the interaction between *GhDSC1* and *GhCAMTA3* and the defence response mediated by both are different in cotton compared to those in *A. thaliana*. These results indicated that the function of GhDSC1 may be coupled with GhCAMTA3 through modulation of JA signalling.

Interestingly, identification of the sequence polymorphism of *GhDSC1* between the resistant and susceptible cotton germplasm accessions showed that a single SNP was responsible for the nonsynonymous mutation in P‐loop motif in *GhDSC1* that, resulted in the conserved glycine residue in resistant germplasm accessions and arginine residue in susceptible germplasm accessions. The NBS domain is mainly involved in ATP hydrolysis (the ADP bound state represents the ‘off’ and the ATP the ‘on’ state) and release of signalling, and the binding activity depends on a functional P‐loop motif, which is a glycine‐rich flexible loop containing a highly conserved lysine residue interacting with the phosphates of the nucleotide and with a magnesium cation that coordinates the β‐ and γ‐phosphates (Belkhadir *et al*., 2004; DeYoung and Innes, 2006; McHale *et al*., 2006; Qi and Innes, 2013). Previous studies showed that mutation of the glycine residue in the P‐loop resulted in the loss of function of several NBS‐LRR genes, such as *RPM1* from *A. thaliana* and *N* from tobacco (Dinesh‐Kumar and Baker, 2000; Tornero *et al*., 2002). In our study, the mutation genotype from susceptible germplasm accessions also showed a reduction in the level of defence responses, including ROS accumulation and regulation of JA signalling‐related genes (Fig. 8E,F,G). In *A. thaliana*, introducing a P‐loop mutation of glycine in multiple *A. thaliana* NBS‐LRR proteins (like the *DSC1*) as the mutation in the susceptible genotype *GhDSC1* in cotton also could disrupt their function in disease resistance (Lolle *et al*., 2017). The mutation in the P‐loop motif may thus underlie the functional divergence of *GhDSC1* between the resistant and susceptible cotton accessions and suggests that the TIR‐NBS‐LRR protein encoded by *GhDSC1 *plays a critical role in Verticillium wilt resistance in cotton.

In conclusion, our study confirmed that *GhDSC1* isolated by the genetic methods, encodes a typical TIR‐NBS‐LRR protein that confers Verticillium wilt resistance by modulating the ROS accumulation and JA signalling‐related genes. In addition, sequence divergence of *GhDSC1* in *G. hirsutum* displayed a nonsynonymous mutation that determines susceptibility or resistance in cotton germplasm accessions. Taken together, our study demonstrated that *GhDSC1* confers Verticillium wilt resistance, and hence is a valuable candidate for breeding wilt resistance in cotton.

## Experimental Procedures

### Plant and fungal culture conditions

The highly virulent *V. dahliae* strain Vd991 (Chen *et al*., 2018) (used in all experiments) was cultured in complete medium (CM) at 25 °C for 5 days on a shaker. Conidia were harvested by centrifugation and washed with sterile water; the final concentration was adjusted to 5 × 10^6^ conidia/mL using a hemocytometer. *A. thaliana* seedlings were grown in pots with potting soil (PINDSTRUP, Denmark) including 20% vermiculite in a greenhouse maintained at 24 °C, 60%–70% relative humidity, and under a 16 h/8 h light/dark photoperiod. Cotton plants were grown and maintained in a greenhouse at 28 °C under a 16 h/8 h light/dark photoperiod. *N. benthamiana *plants were grown at 25 °C for 4 weeks prior to pathogenicity assay and transient expression, under a 14 h/10 h, light/dark regime in greenhouse.

### Gene cloning

To clone *GhDSC1*, 3‐week‐old cotton seedlings of cv. Zhongzhimian No. 2 were inoculated with 5 mL of 5 × 10^6^ conidia/mL conidial suspension, and root samples were collected at 24 h after inoculation. Total RNA was extracted using a Plant RNA Purification Kit (Tiangen, Beijing, China), and cDNA was synthesized by using a RevertAid^TM^ First Strand cDNA Synthesis Kit from MBI (Fermentas, Glen Burnie, Maryland, MA, USA). Primers were designed according to the full ORF of the gene Gh_A10G2076 in the *G. hirsutum* reference genome (Zhang *et al*., 2015) (Table S2). Primers were used to amplify the target fragment from genomic DNA and cDNA. The PCR conditions consisted of an initial 95 °C denaturation step for 10 min, followed by 35 cycles of 95 °C for 30 s, 55 °C for 45 s, and 72 °C for 3 min. PCR products were cloned into the pGEM‐T‐Easy vector (Promega, Madison, WI, USA), transformed into *Escherichia coli* DH5α, and confirmed by sequencing.

### Sequence analyses

The ORFs of *GhDSC1* were determined using ORF Finder (https://www.ncbi.nlm.nih.gov/orffinder/). The conserved domains of *GhDSC1* were predicted using the web‐based programme SMART (Simple Modular Architecture Research Tool, (http://smart.embl.de) (Letunic and Bork, 2018). A phylogenetic tree was constructed using GhDSC1 and the sequences of other known NBS‐LRR resistance associated proteins by Mega 6.0 with Jones‐Taylor‐Thornton model, using maximum‐likelihood with 1000 bootstrap replicates (Tamura *et al*., 2013). Sequence characteristics of known TIR, NB‐ARC and LRR domains in *GhDSC1 *were analysed by the multiple sequence alignment of the *GhDSC1* to known TIR‐NBS‐LRR proteins using the ClustalX 1.83 software (Thompson *et al*., 1997). LRR (L, M and N regions) searches were conducted using the web‐based programme LRRfinder (http://www.lrrfinder.com/lrrfinder.php). The potential subcellular localization of *GhDSC1* was deduced using the web‐based programmes of WolfPsort (https://wolfpsort.hgc.jp/), Signal4.1 (http://www.cbs.dtu.dk/services/SignalP/), THHMM2.0 (http://www.cbs.dtu.dk/services/TMHMM/), and cNLS Mapper (http://nls-mapper.iab.keio.ac.jp/cgi-bin/NLS_Mapper_form.cgi).

### Subcellular localization of *GhDSC1*

To study the subcellular localization of *GhDSC1* *in planta*, and whether the nuclear localization signals (NLS) affect their subcellular localization, the full‐length *GhDSC1* coding region and also the sequences without one or two NLS peptide were inserted into the pBGFP4 vector to generate a C‐terminal fusion with the GFP sequence under the control of Cauliflower mosaic virus (CaMV) 35S promoter, respectively. Plasmids harbouring *GFP* alone (empty vector, *p35S::GFP*) were used as controls. These vectors were transiently expressed in *N. benthamiana* leaves using *Agrobacterium* infection (van der Hoorn *et al*., 2000). The subcellular localization of the above fusion protein was observed 2 days post‐agroinfiltration with a laser scanning confocal microscope (LSMT‐PMT) with excitation at 488 nm and emission at 510 nm. Nuclei were stained with 4',6‐diamidino‐2‐phenylindole (DAPI; Invitrogen, Carlsbad, CA, USA).

### Generation and analysis of transgenic *Arabidopsis thaliana*

The ORF fragments from *GhDSC1 *were amplified with primers containing *Nco*I and *Spe*I enzyme sites and were integrated into the binary vector pCAMBIA1304 under the control of the CaMV35S promoter. The recombinant plasmid (pCAMBIA1304::*GhDSC1*) was transformed into *A. tumefaciens* (strain GV3101) and introduced into 4‐week‐old *A. thaliana *plants (ecotype Col‐0) using an *Agrobacterium*‐mediated floral dip method (Clough and Bent, 1998). Transgenic plants were selected on MS medium containing 50 mg/L hygromycin, and the T_3 _homozygous transgenic plants were identified with PCR and RT‐PCR using genomic DNA and cDNA samples, respectively. The wild‐type gDNA and cDNA were used as controls. The amplification conditions consisted of an initial 95 °C denaturation step for 10 min, which was followed by 35 cycles of 95 °C for 45 s, 58 °C for 30 s, and 72 °C for 1 min; and the gene encoding ubiquitin extension protein 5 (*UBQ5*, NM_116090.3) was used as a control. *GhDSC1* was also introduced into the *A. thaliana GhDSC1* orthologue gene At4g12010.1 (*DSC1*) mutant (*dsc1*, SALK_014299) as described above.

### Detection of transgenic plant resistance to Verticillium wilt

The phenotypes of transgenic *A. thaliana* plants resistant to *V. dahliae *Vd991 were assayed using a root‐dip method. Three‐week‐old *A. thaliana *plants were up‐rooted, and the roots were dipped in *V. dahliae* conidial suspension (5 × 10^6 ^conidia/mL) for 5 mins followed by replanting into vermiculite soil. The Verticillium wilt symptoms were recorded 3 weeks after inoculation.

For fungal biomass quantification, roots and stems of three inoculated plants were harvested at 21 days post‐inoculation. Quantitative PCR was performed using a SYBR Premix Ex Taq II kit (Takara, Japan) with primers for the *V. dahliae *elongation factor 1‐α (*EF‐1α*) and primers for *A. thaliana* *UBQ5* as an endogenous control (Table S2).

### Detection of ROS accumulation

ROS accumulation was detected in transgenic *A. thaliana* and wild‐type (Col‐0) leaves from 3‐week‐old plants 12 h after infiltration with 10 µL of a *V. dahliae* (strain Vd991) conidia suspension (2 × 10^6 ^conidia/mL) using 3'3‐diaminobenzidine (DAB) solution as previously described (Bindschedler *et al*., 2006; Thordal‐Christensen *et al*., 1997). A sterile water treatment was used as the control. For comparing the ROS accumulation after transient expression of the resistance genotype *GhDSC1^R^* and susceptible genotype *GhDSC1^S^*, each was cloned into a PVX vector pCHF3 and transformed into the *A. tumefaciens* strain GV3101. Agroinfiltration assays were performed on *N. benthamiana* plants expressing GFP as a negative control. Four‐week‐old *N. benthamiana* leaves were agroinfiltrated with 10 µL (OD = 0.8) of *GhDSC1^R^* and *GhDSC1^S ^A. tumefaciens* strains, respectively, then conidial suspensions each of 5 × 10^6 ^conidia/mL of *V. dahliae *were inoculated 2 days later. ROS accumulation was stained with DAB solution for detection at 2 days after treatment. Briefly, the leaves were treated with 1 mg/mL DAB containing 0.05% v/v Tween 20 and 10 mM sodium phosphate buffer (pH 7.0). The leaves were incubated at 25 °C in the dark and infiltrated under gentle vacuum. The reaction was terminated at 10 h–12 h post‐inoculation and the DAB solution was removed with a distilled water rinse. Ethanol (75%) was added to the leaves to remove the chlorophyll and placed in 30% glycerol after the decolourization. Six leaves per treatment were included in each of the three replicates. Samples were observed using a SMZ18 stereo microscope (Nikon, Japan). The percentages of brown pixels were obtained in every image (1 cm^2^) from six leaves examined for each treatment, and replicates of the same size and resolution were included in calculations using ImageJ software (Rasband, 2012).

### Relative gene expression analysis

For relative expression analysis of *GhDSC1 *and *GhCAMTA3 *in cotton plants (*G. hirsutum *cv. Zhongzhimian No. 2, cv. AA085, cv. Junmian No. 1, cv. Jimian No. 11), which differed in resistance to Verticillium wilt, the cotton plants were inoculated with a conidial suspension of 5 × 10^6^ conidia/mL of *V. dahliae* (strain Vd991) using a root‐dip method upon the development of the first euphylla. The inoculated samples were collected at seven time points (2, 6, 12, 24, 48, 72 and 120 h) after treatment, with three seedlings for each sample. For the expression analysis of *GhDSC1 *and *GhCAMTA3 *in cotton after hormone treatment, 4‐week‐old seedlings of *G. hirsutum *cv. Zhongzhimian No. 2 with first euphylla were sprayed with 10 mM MeJA, 10 mM SA, 5 mM ETH, 100 μM ABA, respectively. The inoculated samples were collected at six time points (2, 6, 12, 24, 48 and 72 h) after treatment, with three seedlings for each sample. For detection of the expression of *GhDSC1* in different tissues of *G. hirsutum *cv. Zhongzhimian No. 2, the different tissues (leaf, root, stem, petiole, flower, boll and seed) were collected 72 h after inoculation for RNA extraction. For analysis of MeJA signalling‐associated genes and *GhCAMTA3* expression in different *A. thaliana *transgenic lines (ecotype Col‐0, *GhDSC1 *overexpression transgenic Col‐0 mutants, *dsc1* mutants, and *dsc1* mutants complemented with *GhDSC1*), each were inoculated with 5 × 10^6 ^conidia/mL of *V. dahliae* (strain Vd991) conidia suspension using a root‐dip method. Three root samples from each treatment were collected at 24 h after inoculation.

RT‐qPCR analyses were performed using the SYBR Premix Ex Taq kit (Takara, Kusatsu, Shiga, Japan) and a QuantStudio 6 Flex Real‐Time PCR System (Applied Biosystems, Foster City, CA, USA). The PCR cycling programme included an initial denaturation step at 95 °C for 10 min, followed by 40 cycles of denaturation at 95 °C for 15 s, annealing at 60 °C for 30 s, and extension at 72 °C for 20 s. The *A. thaliana UBQ5* (At3g62250, Paparella *et al*., 2014) and *N. benthamiana actin *(Gui *et al*., 2017) were amplified as endogenous controls using the primer pairs listed in Table S2. All assays were carried out with three independent biological replicates. The relative expression levels of genes were evaluated using the 2^‐∆∆CT^ method (Livak and Schmittgen, 2001).

## Supporting information


**Fig. S1** Cloning of *GhDSC1* from *Gossypium hirsutum*. (A) Amplification of* GhDSC1* by Reverse Transcription‐Polymerase Chain Reaction (RT‐PCR). RNA was isolated from cotton roots of *G. hirsutum* cv. Zhongzhimian No. 2 24 h after inoculation with *V. dahliae* Vd991. *GhDSC1* was amplified by RT‐PCR using the cDNA template (cDNA lane). DNA contamination in the RNA sample was assayed by PCR (RNA lane). The *GhDSC1* structure was determined by amplification using the genomic DNA (DNA lane). (B) Exon and intron boundaries of *GhDSC1* were obtained by comparison of the cDNA sequence to the genomic sequence of *GhDSC1*.

**Fig. S2** Structure‐based multiple sequence alignment of the subdomains in *GhDSC1* to known TIR‐NBS‐LRR proteins. The secondary structure assignments of the known TIR‐NBS‐LRR proteins are underlined. Conserved residues are marked by asterisks. *N. glutinosa NgN* (Genebank ID : AAA50763.1), and *A. thaliana AtRPP5* (Genebank ID : AAF08790.1).

**Fig. S3** Prediction of nuclear localization signals (NLS) in *GhDSC1*. Nuclear localization signals prediction of *GhDSC1 *was conducted using the web‐based programme cNLS mapper (http://nls-mapper.iab.keio.ac.jp/cgi-bin/NLS_Mapper_form.cgi). Peptide sequences in red colour represent two nuclear localization signals (NLS1 and NLS2) in *GhDSC1*.

**Fig. S4** Expression analysis of *GhDSC1* in different cotton tissues. Plants of 3‐week‐old cotton (cv. Zhongzhimian No. 2) were inoculated with a suspension of 5 × 10^6 ^conidia/mL of *V. dahliae *strain Vd991 using a root‐dip method. Different tissue samples (leaf, Root, Stem, Petiole, Flower, Boll and Seed) were collected 72 h after inoculation for RNA isolation and cDNA synthesis. Relative expression analysis of *GhDSC1 *was performed by quantitative Reverse Transcription‐quantitative Polymerase Chain Reaction (RT‐qPCR) using the cotton *18S* gene as a reference. Values represent the averages of three independent biological replicates of three plants each. Error bars represent standard errors. Asterisks (∗∗) represent statistical significance at *P *< 0.01, respectively, according to unpaired Student's *t*‐tests of each of the leaf samples used as control.

**Fig. S5** Validation of positive transformants of *GhDSC1 *transgenic *Arabidopsis thaliana *lines. Polymerase Chain Reaction (PCR) products targeting a fragment of *GhDSC1* amplified from DNA extracted from transgenic lines, (A) *GhDSC1*‐overexpressing transgenic lines of *A. thaliana* ecotype Col‐0 and (C) the *GhDSC1*‐recepient *dsc1* mutants. Reverse transcription‐PCR amplification of *GhDSC1* cDNA in the same transgenic *A. thaliana*, (A) *GhDSC1*‐overexpressing transgenic lines of *A. thaliana* ecotype Col‐0 and (C) the *GhDSC1*‐recepient *dsc1* mutants, *UBQ5 *is shown as a control.

**Fig. S6** Quantification of *GhDSC1* expression in the transgenic line overexpressing *GhDSC1* and the* GhDSC1*‐receipient *dsc1* mutant. The transcript levels of GhDSC1 were detected in 3‐week‐old plants grown in Murashige‐Skoog medium. Relative expression analyses of GhDSC1 using Reverse Transcription‐quantitative Polymerase Chain Reaction (RT‐qPCR) was performed using the comparative threshold 2^‐ΔΔCT^ method, and relative expression was compared with expression levels in the transgenic lines overexpressing *GhDSC1* compared to the *GhDSC1*‐receipient *dsc1* mutant. Values represent averages of three independent biological replicates of three plants each. Error bars (standard errors of the mean) were calculated based on three biological replicates using standard deviation.

**Fig. S7** Quantification of *GhCAMTA3* expression in response to ethylene (ETH), salicylic acid (SA) and abscisic acid (ABA) treatment. The transcript levels of *GhCAMTA3* were detected in 3‐week‐old cotton plants (cv. Zhongzhimian No. 2) that treated with the ETH, SA and ABA. Relative expression analyses of *GhCAMTA3* using Reverse rTanscription‐quantitative Polymerase Chain Reaction (RT‐qPCR) was performed using the cotton *18S* gene as a reference using the comparative threshold 2^‐ΔΔCT^ method, and relative expression was compared with expression levels in cotton plants that were treated with sterile water (Mock). Values represent averages of three independent biological replicates of three plants each. Error bars were calculated based on three biological replicates using standard deviation; asterisks (∗) and (∗∗) represent statistical significance at *P *< 0.05 and *P *< 0.01, respectively, according to unpaired Student's *t*‐tests of each of the treatment groups compared to control group (Mock).

**Fig. S8** Nucleotide sequence alignment of *GhDSC1 *in *Gossypium hirsutum *resistant and susceptible germplasm accessions. The alignment was performed by Clustal X2 with a GONNET 80 protein weight matrix. Only residues that deviate from the reference sequences are shown in the alignment; deletions are indicated by dashes (‐). The polymorphism positions are written vertically, i.e. the first polymorphism occurs at position 177 of the CDS. The position in orange colour (673 bp) represents the nonsynonymous mutation in *GhDSC1*.

**Fig. S9** Yeast two‐hybrid assay of *GhDSC1* and *GhCAMTA3* proteins. SD/‐LWHA represents the selection medium lacking Leu, Trp, His and Ade. The interaction of murine p53 (p53) and SV40 large T‐antigen (T) was used as a positive control for the system, and human lamin C (lam) was used in the negative interaction control.

**Table S1** Information of cotton varieties used in this study.

**Table S2** Primers used in this study.
