# Supplementary material for: The Gossypium hirsutum TIR‐NBS‐LRR gene GhDSC1 mediates resistance against Verticillium wilt
Source: Mol Plant Pathol. 2019 Apr 8;20(6):857–76. doi: 10.1111/mpp.12797 (PMC6637886; doi:10.1111/mpp.12797)
Supplement: Supplementary file 1 — Fig. S1 Cloning of GhDSC1 from Gossypium hirsutum. (A) Amplification of GhDSC1 by Reverse Transcription‐Polymerase Chain Reaction (RT‐PCR). RNA was isolated from cotton roots of G. hirsutum cv. Zhongzhimian No. 2 24 h after inoculation with V. dahliae Vd991. GhDSC1 was amplified by RT‐PCR using the cDNA template (cDNA lane). DNA contamination in the RNA sample was assayed by PCR (RNA lane). The GhDSC1 structure was determined by amplification using the genomic DNA (DNA lane). (B) Exon and intron boundaries of GhDSC1 were obtained by comparison of the cDNA sequence to the genomic sequence of GhDSC1. [file MPP-20-857-s001.pdf]

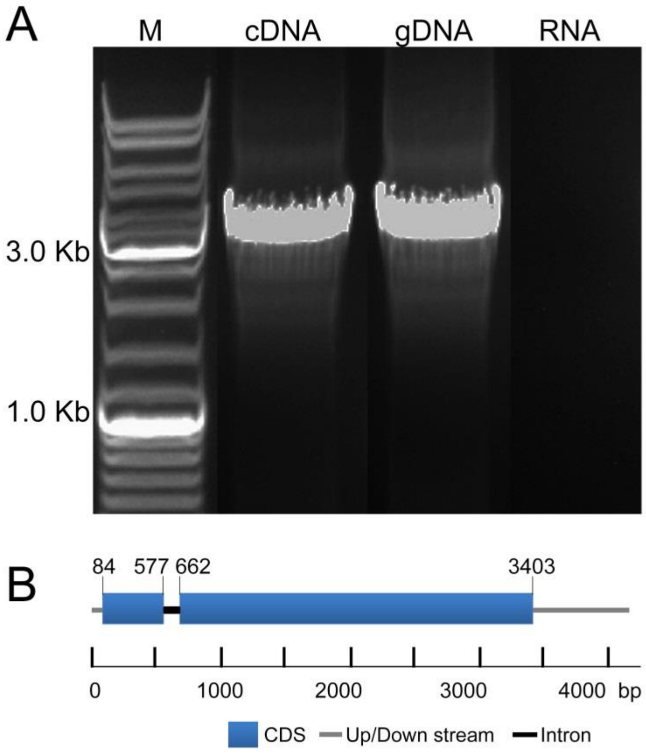

Figure S1 | Cloning of *GhDSC1* from *Gossypium hirsutum*. (**A**) Amplification of *GhDSC1* by reverse transcription-PCR. RNA was isolated from cotton roots of *G. hirsutum* cv. Zhongzhimian No. 2 24 h after inoculation with *V. dahliae* Vd991. After RNA isolation and cDNA synthesis, *GhDSC1* was amplified by RT-PCR using the cDNA template (cDNA lane). DNA contamination in the RNA sample was assayed by PCR (RNA lane). The *GhDSC1* structure was determined by amplification using the genomic DNA (DNA lane). (**B**) The exon and intron boundaries of *GhDSC1* were obtained by comparison the cDNA sequence to the genome sequence of the *Gossypium hirsutum*.
