# Supplementary material for: The Gossypium hirsutum TIR‐NBS‐LRR gene GhDSC1 mediates resistance against Verticillium wilt
Source: Mol Plant Pathol. 2019 Apr 8;20(6):857–76. doi: 10.1111/mpp.12797 (PMC6637886; doi:10.1111/mpp.12797)
Supplement: Supplementary file 2 — Fig. S2 Structure‐based multiple sequence alignment of the subdomains in GhDSC1 to known TIR‐NBS‐LRR proteins. The secondary structure assignments of the known TIR‐NBS‐LRR proteins are underlined. Conserved residues are marked by asterisks. N. glutinosa NgN (Genebank ID : AAA50763.1), and A. thaliana AtRPP5 (Genebank ID : AAF08790.1). [file MPP-20-857-s002.pdf]

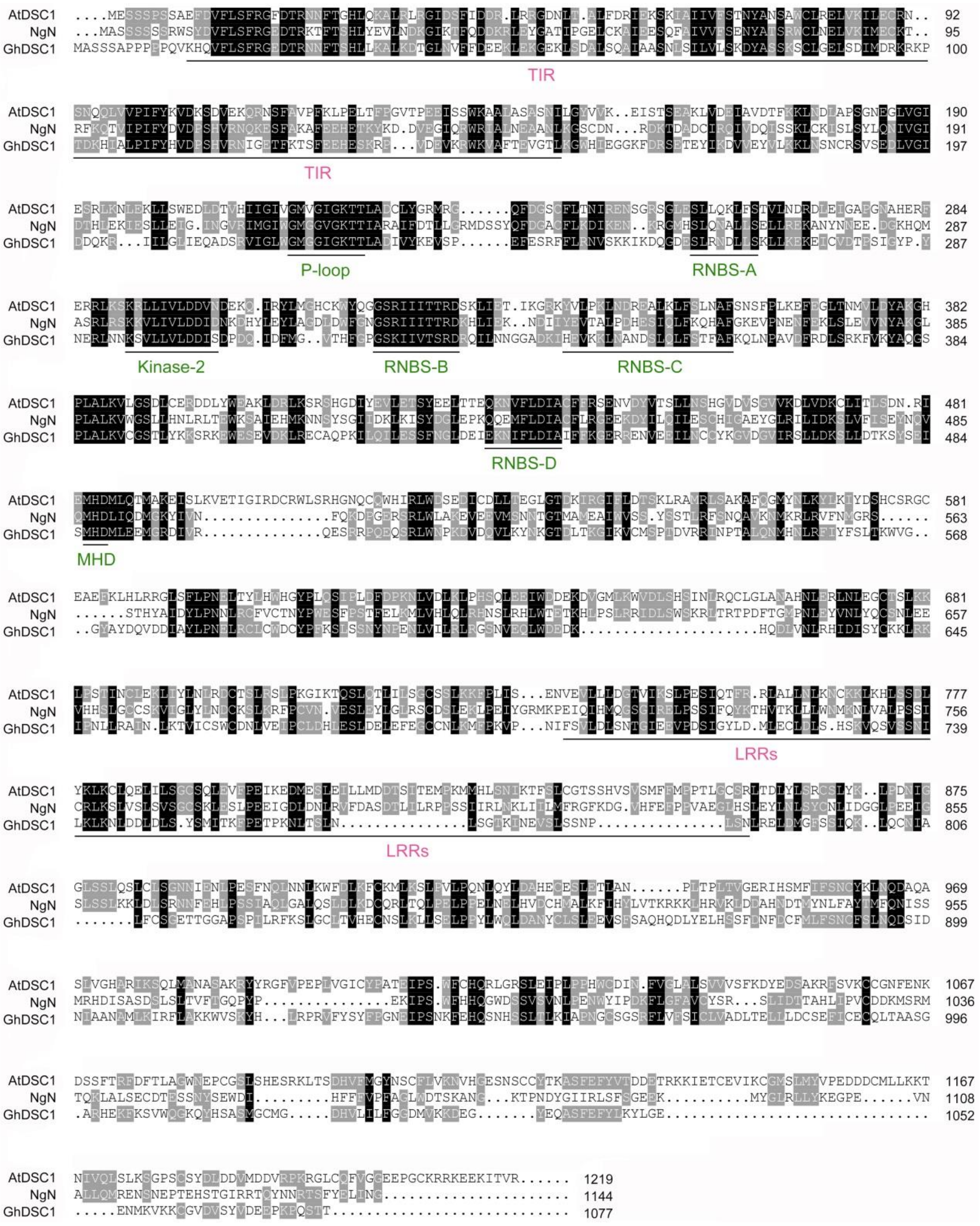

Figure S2 | Structure-based multiple sequence alignment of the subdomains in *GhDSC1* to known TIR-NBS-LRR proteins. The secondary structure assignments of the known TIR-NBS-LRR proteins are underlined. Conserved residues are marked by asterisks. *N. glutinosa NgN* (Genebank ID: AAA50763.1), and *A. thaliana AtRPP5* (Genebank ID: AAF08790.1).
