# Supplementary material for: The Gossypium hirsutum TIR‐NBS‐LRR gene GhDSC1 mediates resistance against Verticillium wilt
Source: Mol Plant Pathol. 2019 Apr 8;20(6):857–76. doi: 10.1111/mpp.12797 (PMC6637886; doi:10.1111/mpp.12797)
Supplement: Supplementary file 3 — Fig. S3 Prediction of nuclear localization signals (NLS) in GhDSC1. Nuclear localization signals prediction of GhDSC1 was conducted using the web‐based programme cNLS mapper (http://nls-mapper.iab.keio.ac.jp/cgi-bin/NLS_Mapper_form.cgi). Peptide sequences in red colour represent two nuclear localization signals (NLS1 and NLS2) in GhDSC1. [file MPP-20-857-s003.pdf]

**Predicted NLSs in query sequence**

```
          NLS1
MASSSAPPPPPQVKHQVFLSFRGEDTRNNFTSHLLKALKDTGLNVFFDEE  50
KLEKGEKLSDALSQAIAASNLSILVLSKDYASSKSCLGELSDIMDRKRKP  100
TDKHIALPIFYHVDPSHVRNIGETFKTSFEEHESKRPVDEVKRWKVAFTE  150
VGTLKGWHIEGGKFDRSETEYIKDVVEYVLKKLNSNCRSVSEDLVGIDDQ  200
KRIILGLIEQADSRVIGLWGMGGIGKTTLADIVYKEVSPEFESRFFLRNV  250
SKKIKDQGDESLRNDLLSKLLKEKEICVDTPSIGYPYNERLNNKSVLLVL  300
DDISDPDQIDFMGVTHFGPGSKIIVTSRDRQILNNGGADKIHEVKKLNAN  350
DSLQLFSTFAFKQLNPAVDFRDLSRKFVKYAQGSPLALKVCGSTLYKKSR  400
KEWESEVDKLRECAQPKILQILESSFNGLDEIEKNIFLDIAIFFKGERRE  450
NVEEILNCCYKGVDGVIRSLLDKSLLDTKSYSEISMHDMLEEMGRDIVRQ  500
ESRRPQEQSRLWNPKDVDQVLKYNKGTDLTKGIKVCMSPTDVRRINPTAL  550
QNMHNLRFIYFSLTKWVGGYAYDQVDDIAYLPNELRCLCWDCYPFKSLSS  600
NYNPENLVILRLRGSNVEQLWDEDKHQDLVNLRHIDISYCKKLRKIPNLL  650
RAINLKTVICSWCDNLVEIPCLDHLESLDELEFEGCCNLKMFPKVPNIFS  700
VLDLSNTGIEEVPDSIGYLDMLECLDLSHSKVQSVSSNILKLKNLDDLDL  750
SYSMITKFPETPKNLTSLNLSGTKINEVSLSSNPLSNLRELDMGFSSIQK  800
LQCNIALFCSGETTGGAPSPILRFKSLGCLTVHECNSLKLLSELPPYLWQ  850
LDANYCLSLEEVSFSAQHQDLYELHSSFDNFDCFMLFSNCFSLNQDSIDN  900
IAANAMLKIRFLAKKWVSKYHLRPRVFYSYFPGNEIPSNKFEHQSNHSSL  950
TLKIAPNGCSGSRFLVFSICLVADLTELLLDCSEFICECQLTAASGARHE  1000
KFKSVWQGKQYHSASMGCMGDHVLILFGGDMVKKDEGYEQASFEFYLKYL  1050
GEENMKVKKCGVDVSYVDEEPKPQSTT     NLS2                1077
```

**Predicted bipartite NLS**

| Pos. | Sequence | Score |
|---|---|---|
| 11 | PQVKHQVFLSFRGEDTRNNFTSHLLKALKDT | 6.9 |
| 1030 | DMVKKDEGYEQASFEFYLKYLGEENMKVKKC | 5.7 |

**Figure S3 | Prediction of nuclear localization signals in *GhDSC1*.** Nuclear localization signals prediction of *GhDSC1* was conducted using the web-based program cNLS mapper (http://nls-mapper.iab.keio.ac.jp/cgi-bin/NLS_Mapper_form.cgi). Peptide sequences in red color represent two nuclear localization signals (NLS1 and NLS2) in *GhDSC1*.
