# Supplementary material for: The Gossypium hirsutum TIR‐NBS‐LRR gene GhDSC1 mediates resistance against Verticillium wilt
Source: Mol Plant Pathol. 2019 Apr 8;20(6):857–76. doi: 10.1111/mpp.12797 (PMC6637886; doi:10.1111/mpp.12797)
Supplement: Supplementary file 4 — Fig. S4 Expression analysis of GhDSC1 in different cotton tissues. Plants of 3‐week‐old cotton (cv. Zhongzhimian No. 2) were inoculated with a suspension of 5 × 10^6^ conidia/mL of V. dahliae strain Vd991 using a root‐dip method. Different tissue samples (leaf, Root, Stem, Petiole, Flower, Boll and Seed) were collected 72 h after inoculation for RNA isolation and cDNA synthesis. Relative expression analysis of GhDSC1 was performed by quantitative Reverse Transcription‐quantitative Polymerase Chain Reaction (RT‐qPCR) using the cotton 18S gene as a reference. Values represent the averages of three independent biological replicates of three plants each. Error bars represent standard errors. Asterisks (∗∗) represent statistical significance at P < 0.01, respectively, according to unpaired Student's t‐tests of each of the leaf samples used as control. [file MPP-20-857-s004.pdf]

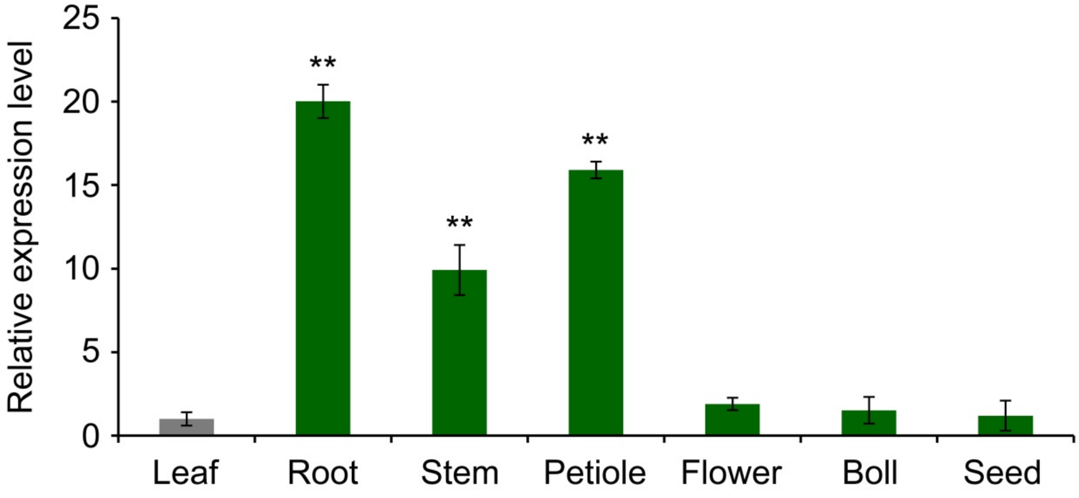

Figure S4 | Expression analysis of *GhDSC1* in different cotton tissues. The adult-plant stage of cotton plants (cv. Zhongzhimian No.2) were irrigated with a suspension of 5×10^6 conidia/mL of *V. dahliae* strain Vd991. Different tissue samples (leaf, Root, Stem, Petiole, Flower, Boll and Seed) were collected 72 hours after inoculation for RNA isolation and cDNA synthesis. Relative expression analysis of *GhDSC1* was performed by reverse transcription-quantitative PCR using the cotton *18S* gene as a reference. Values represent the averages of three independent biological replicates of three plants each. Error bars represent standard errors. Asterisks (∗∗) represent statistical significance of $P < 0.01$, respectively, according to an unpaired Student's *t*-tests of each of the leaf samples used as a control.
