# Supplementary material for: The Gossypium hirsutum TIR‐NBS‐LRR gene GhDSC1 mediates resistance against Verticillium wilt
Source: Mol Plant Pathol. 2019 Apr 8;20(6):857–76. doi: 10.1111/mpp.12797 (PMC6637886; doi:10.1111/mpp.12797)
Supplement: Supplementary file 5 — Fig. S5 Validation of positive transformants of GhDSC1 transgenic Arabidopsis thaliana lines. Polymerase Chain Reaction (PCR) products targeting a fragment of GhDSC1 amplified from DNA extracted from transgenic lines, (A) GhDSC1‐overexpressing transgenic lines of A. thaliana ecotype Col‐0 and (C) the GhDSC1‐recepient dsc1 mutants. Reverse transcription‐PCR amplification of GhDSC1 cDNA in the same transgenic A. thaliana, (A) GhDSC1‐overexpressing transgenic lines of A. thaliana ecotype Col‐0 and (C) the GhDSC1‐recepient dsc1 mutants, UBQ5 is shown as a control. [file MPP-20-857-s005.pdf]

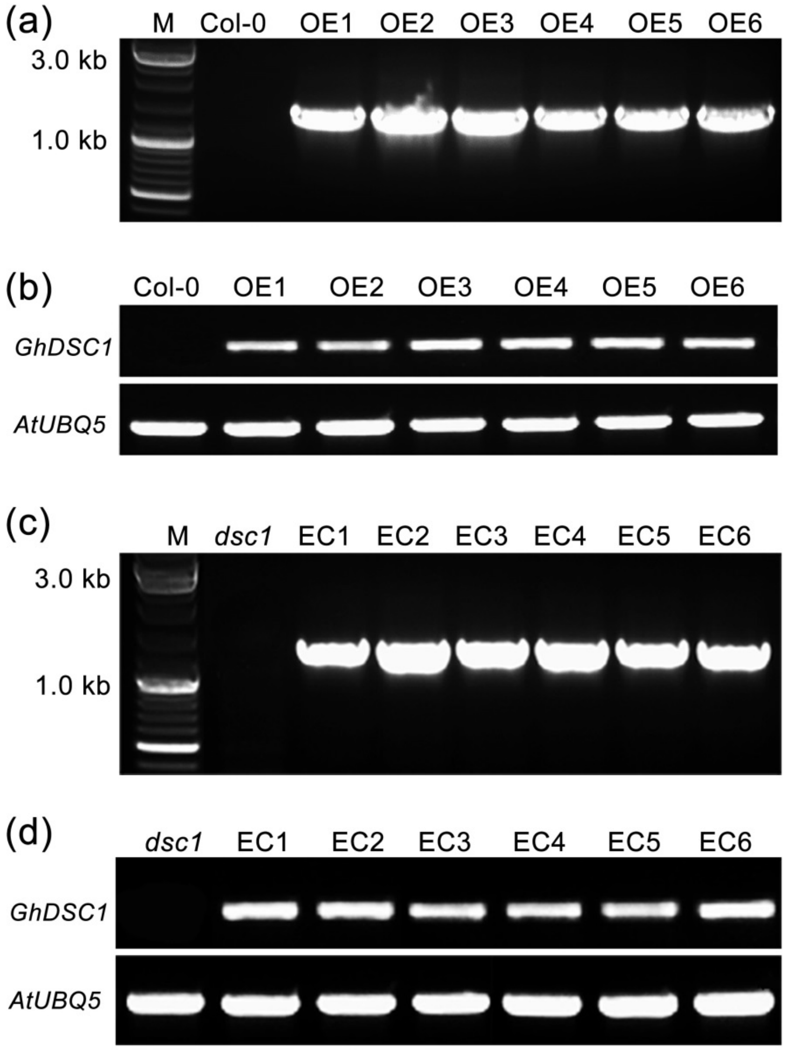

Figure S5 | Validation of positive transformants of *GhDSC1* transgenic *Arabidopsis thaliana* lines. (**A**) and (**C**) PCR products targeting a fragment of *GhDSC1* amplified from DNA extracted from *GhDSC1*-overexpressing transgenic lines of *A. thaliana* ecotype Col-0 (OE) and *A. thaliana dsc1* mutant introduction of *GhDSC1* (EC). (**B**) and (**D**) Reverse transcription-PCR amplification of *GhDSC1* cDNA in the same transgenic *A. thaliana*. *UBQ5* is shown as a control.
