# Supplementary material for: The Gossypium hirsutum TIR‐NBS‐LRR gene GhDSC1 mediates resistance against Verticillium wilt
Source: Mol Plant Pathol. 2019 Apr 8;20(6):857–76. doi: 10.1111/mpp.12797 (PMC6637886; doi:10.1111/mpp.12797)
Supplement: Supplementary file 6 — Fig. S6 Quantification of GhDSC1 expression in the transgenic line overexpressing GhDSC1 and the GhDSC1‐receipient dsc1 mutant. The transcript levels of GhDSC1 were detected in 3‐week‐old plants grown in Murashige‐Skoog medium. Relative expression analyses of GhDSC1 using Reverse Transcription‐quantitative Polymerase Chain Reaction (RT‐qPCR) was performed using the comparative threshold 2‐ΔΔCT method, and relative expression was compared with expression levels in the transgenic lines overexpressing GhDSC1 compared to the GhDSC1‐receipient dsc1 mutant. Values represent averages of three independent biological replicates of three plants each. Error bars (standard errors of the mean) were calculated based on three biological replicates using standard deviation. [file MPP-20-857-s006.pdf]

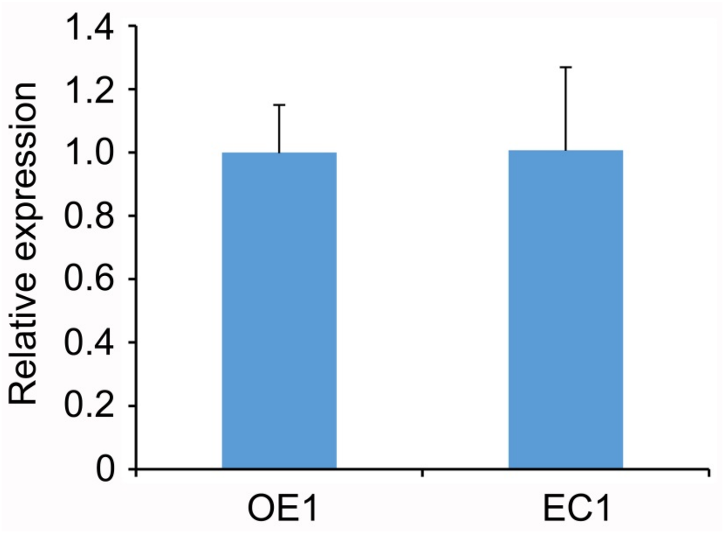

Figure S6 | Quantification of *GhDSC1* expression in the transgenic line overexpressing *GhDSC1* and the *GhDSC1*-receipient *dsc1* mutant. The transcript levels of GhDSC1 was detected in three-week-old plants that growth in Murashige-Skoog medium. Relative expression analyses of *GhDSC1* using reverse transcription-quantitative PCR (RT-qPCR) was performed using the comparative threshold $2^{-\Delta\Delta CT}$ method, and relative expression was compared with expression levels in the transgenic lines overexpressing *GhDSC1* compared to the *GhDSC1*-receipient *dsc1* mutant. Values represent averages of three independent biological replicates of three plants each. Error bars were calculated based on three biological replicates using standard deviation.
