# Supplementary material for: The Gossypium hirsutum TIR‐NBS‐LRR gene GhDSC1 mediates resistance against Verticillium wilt
Source: Mol Plant Pathol. 2019 Apr 8;20(6):857–76. doi: 10.1111/mpp.12797 (PMC6637886; doi:10.1111/mpp.12797)
Supplement: Supplementary file 7 — Fig. S7 Quantification of GhCAMTA3 expression in response to ethylene (ETH), salicylic acid (SA) and abscisic acid (ABA) treatment. The transcript levels of GhCAMTA3 were detected in 3‐week‐old cotton plants (cv. Zhongzhimian No. 2) that treated with the ETH, SA and ABA. Relative expression analyses of GhCAMTA3 using Reverse rTanscription‐quantitative Polymerase Chain Reaction (RT‐qPCR) was performed using the cotton 18S gene as a reference using the comparative threshold 2‐ΔΔCT method, and relative expression was compared with expression levels in cotton plants that were treated with sterile water (Mock). Values represent averages of three independent biological replicates of three plants each. Error bars were calculated based on three biological replicates using standard deviation; asterisks (∗) and (∗∗) represent statistical significance at P < 0.05 and P < 0.01, respectively, according to unpaired Student's t‐tests of each of the treatment groups compared to control group (Mock). [file MPP-20-857-s007.pdf]

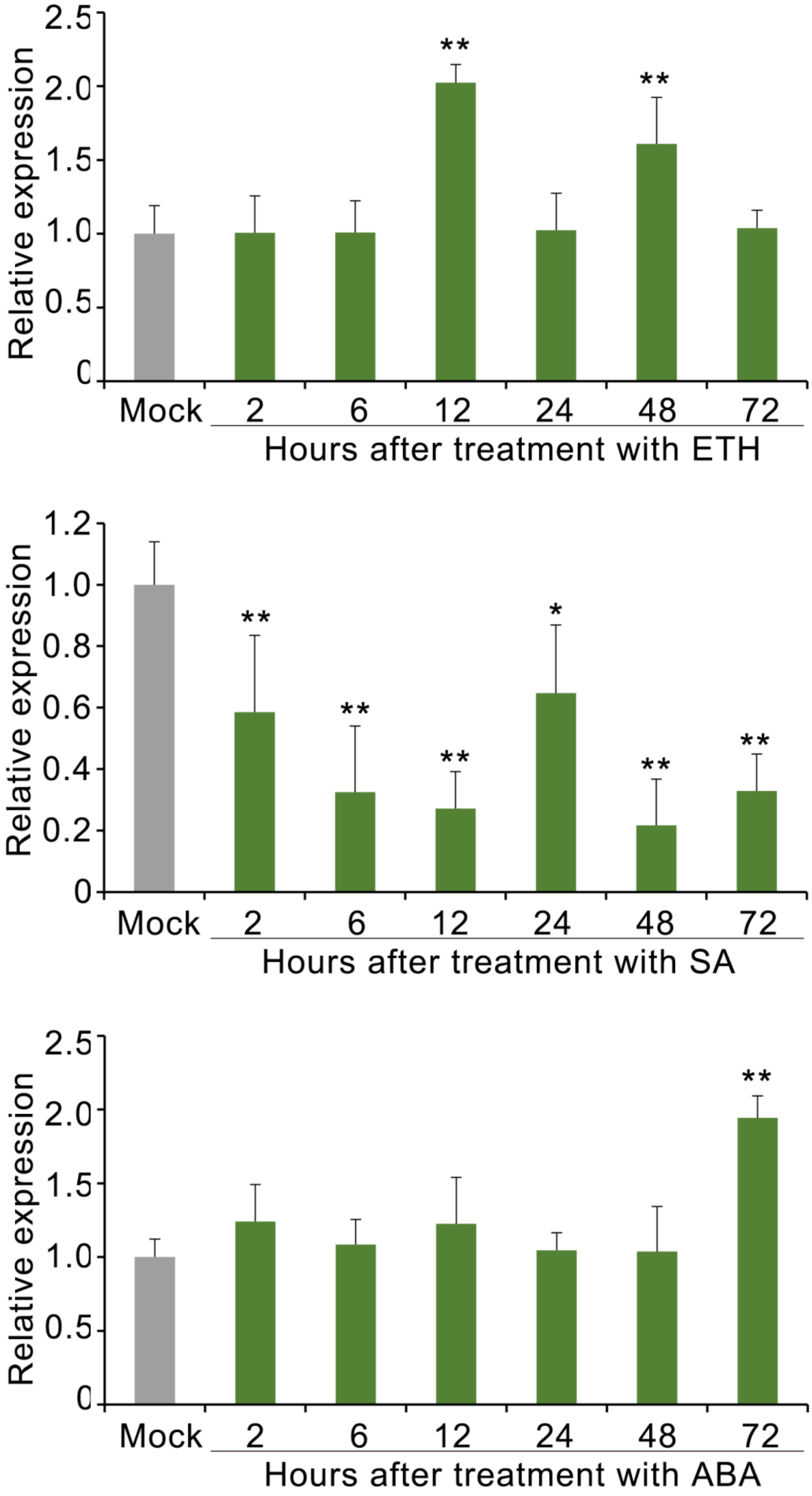

Figure S7 | Quantification of *GhCAMTA3* expression in response to ethylene (ETH), salicylic acid (SA), and abscisic acid (ABA) treatment. The transcript

levels of *GhCAMTA3* were detected in three-week-old cotton plants (cv. Zhongzhimian No. 2) that treated with the ETH, SA, and ABA. Relative expression analyses of *GhCAMTA3* using reverse transcription-quantitative RT-qPCR was performed using the cotton *18S* gene as a reference using the comparative threshold $2^{-\Delta\Delta CT}$ method, and relative expression was compared with expression levels in cotton plants that were treated with sterile water (Mock). Values represent averages of three independent biological replicates of three plants each. Error bars were calculated based on three biological replicates using standard deviation; asterisks (∗) and double asterisks (∗∗) represents statistical significance of $P < 0.05$ and $P < 0.01$, respectively, according to an unpaired Student's t-tests of each of the treatment groups compared to control group (Mock).
