# Supplementary material for: The Gossypium hirsutum TIR‐NBS‐LRR gene GhDSC1 mediates resistance against Verticillium wilt
Source: Mol Plant Pathol. 2019 Apr 8;20(6):857–76. doi: 10.1111/mpp.12797 (PMC6637886; doi:10.1111/mpp.12797)
Supplement: Supplementary file 8 — Fig. S8 Nucleotide sequence alignment of GhDSC1 in Gossypium hirsutum resistant and susceptible germplasm accessions. The alignment was performed by Clustal X2 with a GONNET 80 protein weight matrix. Only residues that deviate from the reference sequences are shown in the alignment; deletions are indicated by dashes (‐). The polymorphism positions are written vertically, i.e. the first polymorphism occurs at position 177 of the CDS. The position in orange colour (673 bp) represents the nonsynonymous mutation in GhDSC1. [file MPP-20-857-s008.pdf]

Phenotype	Positions / Germplasm	7<br>7<br>1	6<br>8<br>4	9<br>6<br>6	3<br>7<br>6	4<br>4<br>3<br>1	2<br>4<br>4<br>2	8<br>6<br>6<br>2	9<br>6<br>6<br>2	0<br>7<br>6<br>2	1<br>7<br>6<br>2	9<br>1<br>9<br>2
Resistance	Zhongzhimian 2	A	C	T	G	A	A	T	G	C	T	T
	Kelin 15-416	A	C	T	G	A	T	T	G	C	T	G
	Emian 23	A	C	T	G	A	T	T	G	C	T	A
	20040701	T	G	T	G	A	A	T	G	C	T	T
	Jimian 616	A	C	A	C	T	A	T	G	C	T	A
	Zhongmiansuo 60	A	C	T	G	A	A	T	G	C	T	A
	Yangpin 2	A	C	T	G	A	A	-	-	-	T	T
	TZ06	A	C	T	G	A	A	T	G	C	T	A
	Chuangmian36	A	C	T	G	A	A	T	G	C	T	T
Susceptible	Jimian 11	A	G	T	C	A	T	T	G	C	T	C
	Liaomian 30	A	G	A	C	T	A	T	G	C	T	C
	XZ-14	A	G	A	C	T	T	T	G	C	T	T
	Hui 30	A	C	A	C	T	A	T	G	C	T	A
	Han8944	A	C	A	C	T	A	T	-	-	-	T
	Xinluzao 39	A	G	A	C	T	A	T	G	C	T	G
	Junmian 1	A	C	A	C	T	A	T	G	C	T	T
	Jiumian 11	A	C	A	C	T	T	T	G	C	T	T
	Su22	T	G	A	C	T	A	T	G	C	T	A

Figure S8 | Nucleotide sequence alignment of *GhDSC1* in *Gossypium hirsutum* resistant and susceptible germplasm accessions. The alignment was performed by Clustal X2 with a GONNET 80 protein weight matrix. Only residues that deviate from the reference sequences are shown in the alignment; deletions are indicated by dashes (-). The polymorphism positions are written vertically, i.e. the first polymorphism occurs at position 177 of the CDS. The position in orange color (673 bp) represent the nonsynonymous mutation in *GhDSC1*.
