# Supplementary material for: The Gossypium hirsutum TIR‐NBS‐LRR gene GhDSC1 mediates resistance against Verticillium wilt
Source: Mol Plant Pathol. 2019 Apr 8;20(6):857–76. doi: 10.1111/mpp.12797 (PMC6637886; doi:10.1111/mpp.12797)
Supplement: Supplementary file 9 — Fig. S9 Yeast two‐hybrid assay of GhDSC1 and GhCAMTA3 proteins. SD/‐LWHA represents the selection medium lacking Leu, Trp, His and Ade. The interaction of murine p53 (p53) and SV40 large T‐antigen (T) was used as a positive control for the system, and human lamin C (lam) was used in the negative interaction control. [file MPP-20-857-s009.pdf]

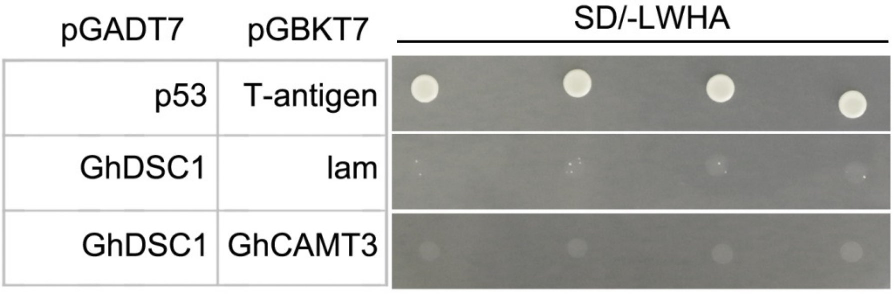

Figure S9 | Yeast two-hybrid assay of *GhDSC1* and *GhCAMTA3* proteins. SD/-LWHA represents the selection medium lacking Leu, Trp, His, and Ade. The interaction of murine p53 (p53) and SV40 large T-antigen (T) was used as a positive control for the system, and human lamin C (lam) was used in the negative interaction control.
