# Supplementary material for: The Gossypium hirsutum TIR‐NBS‐LRR gene GhDSC1 mediates resistance against Verticillium wilt
Source: Mol Plant Pathol. 2019 Apr 8;20(6):857–76. doi: 10.1111/mpp.12797 (PMC6637886; doi:10.1111/mpp.12797)
Supplement: Supplementary file 10 — Table S1 Information of cotton varieties used in this study. [file MPP-20-857-s010.pdf]

Table S1 Information of cotton varieties used in this study

Species	Genotype	Code ID	Name in this study	Origin
Gossypium hirsutum	Resistance	A240	Zhongzhimian 2	Beijing, China
Gossypium hirsutum	Resistance	A238	Kelin 15-416	Henan, China
Gossypium hirsutum	Resistance	A013	Emian 23	Hubei, China
Gossypium hirsutum	Resistance	A180	20040701	Hubei, China
Gossypium hirsutum	Resistance	A222	Jimian 616	Hebei, China
Gossypium hirsutum	Resistance	A277	Zhongmiansuo 60	Henan, China
Gossypium hirsutum	Resistance	A233	Yangpin 2	Hebei, China
Gossypium hirsutum	Resistance	A081	TZ06	Hubei, China
Gossypium hirsutum	Resistance	A095	Chuanmian 36	Sichuan, China
Gossypium hirsutum	Susceptible	A290	Jimian 11	Hebei, China
Gossypium hirsutum	Susceptible	A103	Liaomian 30	Liaoning, China
Gossypium hirsutum	Susceptible	A025	XZ-14	Hunan, China
Gossypium hirsutum	Susceptible	A076	Hui 30	Hubei, China
Gossypium hirsutum	Susceptible	A095	chuanmian 36	Hebei, China
Gossypium hirsutum	Susceptible	A203	Xinluzao 39	Xinjiang, China
Gossypium hirsutum	Susceptible	A293	Junmian 1	Xinjiang, China
Gossypium hirsutum	Susceptible	A141	Jiumian 11	Gansu, China
Gossypium hirsutum	Susceptible	A010	Su22	Jiangsu, China
