# Supplementary material for: The Gossypium hirsutum TIR‐NBS‐LRR gene GhDSC1 mediates resistance against Verticillium wilt
Source: Mol Plant Pathol. 2019 Apr 8;20(6):857–76. doi: 10.1111/mpp.12797 (PMC6637886; doi:10.1111/mpp.12797)
Supplement: Supplementary file 11 — Table S2 Primers used in this study. [file MPP-20-857-s011.pdf]

Table S2 Primers used in this study

Primer name	Primer sequence (5′ -3′)	Purpose
**Full length DNA and cDNA amplify**		
GhDSC1-F-F	AATGAAATGAAACCCAC	Primers for full length amplify of *GhDSC1*
GhDSC1-F-R	AAATCAAAGTTCCCTCA	Primers for full length amplify of *GhDSC1*
**Gene expression pattern analysis**		
GhDSC1-Q-F	TCCTCCTCCTCCTCAAGT	RT-qPCR for *GhDSC1* gene
GhDSC1-Q-R	TGCGATCCATAATGTCAGAA	RT-qPCR for *GhDSC1* gene
GhCAMTA3-Q-F	TTCTTCGTTGCCACACTC	RT-qPCR for *GhCAMTA3* gene
GhCAMTA3-Q-R	ACTCTCGGACTTCACTACA	RT-qPCR for *GhCAMTA3* gene
CAMTA3-Q-F	GCAAGACGGAACAGAGAA	RT-qPCR for CAMTA3 gene
CAMTA3-Q-R	AGGACATAGGCAACATCAAT	RT-qPCR for CAMTA3 gene
**Primers for transgenic lines detection**		
GhDSC1-T-F	TCCTCCTCCTCCTCAAGT	Primers for *GhDSC1* transgenic lines detecting
GhDSC1-T-R	CTCCTTATTACACCATCTACAC	Primers for *GhDSC1* transgenic lines detecting
**Primers for fungal biomass detection**		
VdEF-1α-F	TGAGTTCGAGGCTGGTATCT	For qPCR measurement of *V. dahliae* level
VdEF-1α-R	CACTTGGTGGTGTCCATCTT	For qPCR measurement of *V. dahliae* level
Gh18S-F	CGGCTACCACATCCAAGGAA	Endogenous control for qPCR in *G. hirsutum*
Gh18S-R	TGTCACTACCTCCCCGTGTCA	Endogenous control for qPCR in *G. hirsutum*
**Expression detection of marker genes ralated to JA pathway in *A. thaliana***		
AtPDF1.2-Q-F	CTTGTTCTCTTTGCTGCTTTC	RT-qPCR for *AtPDF1.2* gene
AtPDF1.2-Q-R	CATGTTTGGCTCCTTCAAG	RT-qPCR for *AtPDF1.2* gene
AtVSP2-Q-F	TCATACTCAGTGACCGTTGG	RT-qPCR for *AtVSP2 g*ene
AtVSP2-Q-R	TGTACACCACTTGCCTCAAG	RT-qPCR for *AtVSP2 g*ene
AtPR3-Q-F	CGGCGACCTCCTTCTTCCTC	RT-qPCR for *AtPR3* gene
AtPR3-Q-R	GAATGATGCCGCTTGTCCTG	RT-qPCR for *AtPR3* gene
AtPR4-Q-F	AATGGATCCACAATGCGGTCGTCA	RT-qPCR for *AtPR4* gene
AtPR4-Q-R	AATGAATTCTTCTGGAATAGGCTG	RT-qPCR for *AtPR4* gene
AtUBQ5-Q-F	GACGCTTCATCTCGTCC	Endogenous control for RT-qPCR in *A. thaliana*
AtUBQ5-Q-R	GTAAACGTAGGTGAGTCCA	Endogenous control for RT-qPCR in *A. thaliana*
**Expression detection of marker genes ralated to JA pathway in *N. benthamiana***		
NbPDF1.2-Q-F	ATCTGTCTGGGGAAATGGCA	RT-qPCR for *NbPDF1.2* gene
NbPDF1.2-Q-R	CATGGTCCCTTGAAACGGTG	RT-qPCR for *NbPDF1.2* gene
NbVSP2-Q-F	TCATACTCAGTGACCGTTGG	RT-qPCR for *NbVSP2* gene
NbVSP2-Q-R	TGTACACCACTTGCCTCAAG	RT-qPCR for *NbVSP2* gene
NbPR3-Q-F	CACCAGGACTAACACCAAG	RT-qPCR for *NbPR3* gene
NbPR3-Q-R	CTCTCCTACTCCTCTCTGC	RT-qPCR for *NbPR3* gene
NbPR4-Q-F	TGGGTGGACATATTACAGAG	RT-qPCR for *NbPR4* gene
NbPR4-Q-R	GGCACGCCGACACATTT	RT-qPCR for *NbPR4* gene
NbActin-Q-F	CGAGGGTTATGCTTTGCCTC	Endogenous control for RT-qPCR in *N. benthamiana*
NbActin-Q-R	AGCAAGCTCCTCCTTCATGT	Endogenous control for RT-qPCR in *N. benthamiana*
